# A systems genetic analysis identifies putative mechanisms and candidate genes regulating vessel traits in poplar wood

**DOI:** 10.3389/fpls.2024.1375506

**Published:** 2024-05-29

**Authors:** F. Daniela Rodriguez-Zaccaro, Meric Lieberman, Andrew Groover

**Affiliations:** ^1^ USDA Forest Service, Pacific Southwest Research Station, Davis, CA, United States; ^2^ University of California Davis, Genome Center, Davis, CA, United States; ^3^ USDA Forest Service, Northern Research Station, Burlington, VT, United States

**Keywords:** drought, forest tree, forest management, *populus*, water transport, wood development

## Abstract

Wood is the water conducting tissue of tree stems. Like most angiosperm trees, poplar wood contains water-conducting vessel elements whose functional properties affect water transport and growth rates, as well as susceptibility to embolism and hydraulic failure during water stress and drought. Here we used a unique hybrid poplar pedigree carrying genomically characterized chromosomal insertions and deletions to undertake a systems genomics analysis of vessel traits. We assayed gene expression in wood forming tissues from clonal replicates of genotypes covering dosage quantitative trait loci with insertions and deletions, genotypes with extreme vessel trait phenotypes, and control genotypes. A gene co-expression analysis was used to assign genes to modules, which were then used in integrative analyses to identify modules associated with traits, to identify putative molecular and cellular processes associated with each module, and finally to identify candidate genes using multiple criteria including dosage responsiveness. These analyses identified known processes associated with vessel traits including stress response, abscisic acid and cell wall biosynthesis, and in addition identified previously unexplored processes including cell cycle and protein ubiquitination. We discuss our findings relative to component processes contributing to vessel trait variation including signaling, cell cycle, cell expansion, and cell differentiation.

## Introduction

Wood development is a culmination of cell division, tissue patterning and cell differentiation that is modified to balance growth versus safety under environmental conditions that vary throughout growing seasons and across years. The vascular cambium is a secondary meristem composed of ray and fusiform initials that give rise to both wood and inner bark tissues (Larson, 1994). In the developing wood of the model tree genus *Populus*, fusiform initials give rise to the axial cell system that includes fibers and vessel elements within wood. Vessel elements, the water conducting cells in wood, develop through rapid and dramatic radial cell expansion involving both symplastic and intrusive growth (Wilczek et al., 2011). After expansion, vessel elements synthesize a complex lignified secondary cell wall, followed by program cell death and hydrolysis of the cytoplasmic contents and portions of the primary cell wall (Turner et al., 2007). Dead at functional maturity, individual vessel elements connect end-to-end to form longer conduits called vessels (Esau, 1977), allowing for the upward flow of water under tension. The dimensions and distribution of vessels in wood help determine tissue hydraulic and mechanical functions (Sperry, 2011).

The width, number, and clustering of vessels affect water transport efficiency and vulnerability to hydraulic failure under stressful conditions (Rodriguez-Zaccaro and Groover, 2019). Stressful environmental conditions including drought, extreme heat and high salinity, reduce cambial activity and result in wood with vessels that are smaller in diameter, more numerous and more interconnected (Arend and Fromm, 2007; Fonti et al., 2013). Small variations in vessel diameter can have a large impact on water transport efficiency, with wider vessels allowing for greater volumetric flow rates (Baas et al., 2004; Venturas et al., 2017). Vessels that are in direct contact share pit pairs through which water can flow laterally. Consequently, increased vessel clustering (measured through vessel grouping indices) allows for greater vessel network interconnectivity and greater stem-specific hydraulic conductivities (Lens et al., 2011; Hajek et al., 2014). The ability of trees to transport water efficiently is tied to their photosynthetic capacity and growth rate (Tyree et al., 1998; Brodribb and Field, 2000). Greater water transport efficiency, however, has been linked to increased vulnerability to hydraulic failure through freeze-thaw and drought-induced air embolisms that can lead to death (Feng et al., 2015; Gleason et al., 2016). Trees are thus challenged to sense environmental conditions and adjust wood development accordingly to balance growth and safety.

A number of vessel traits have been linked to hydraulic properties of tree stems. The number of vessels within a cross-sectional area of wood (termed vessel frequency) is often strongly negatively correlated to vessel diameter (Sperry et al., 2008). Greater vessel frequencies can buffer trees from hydraulic failure by decreasing the impact of embolism of a single vessel on the overall conductivity of a stem (Baas et al., 1983; Ewers et al., 2007). Some studies have linked greater vessel grouping to increased vulnerability to embolism (Scholz et al., 2013; Guet et al., 2015), possibly due to an increased probability of embolism spread throughout a more interconnected xylem network (Loepfe et al., 2007). However, greater vessel grouping indices have also been related to decreased vulnerability to embolism (Lens et al., 2011; Smith et al., 2013), likely due to the presence of alternate pathways that can bypass embolism blockage (Carlquist, 1984). The cross-sectional shape of vessels (termed vessel circularity) can also affect transport efficiency, as deviations from perfect circularity can decrease the volumetric flow rate of water through a conduit (Zhao et al., 2019). Vessel width and frequency scale with tree height and diameter; taller and thicker trees have wider and less numerous vessels than shorter and thinner trees at the same sampling height (Olson et al., 2014; Olson et al., 2020). Accordingly, taller trees, while more conductive, are generally more vulnerable to drought and freeze-thaw cycles (Olson et al., 2018).

Our understanding of the regulation of vessel traits in trees remains incomplete and there has been ongoing debate regarding whether vessel traits are at all directly genetically regulated or if they represent an indirect response to environmental conditions (Olson et al., 2014; Olson et al., 2020). Emerging evidence gives strong support to the hypothesis that changes in wood development and resulting vessel traits are the result of coordinated regulation, however. For example, abscisic acid plays a key role in the mediation of stress response signals in poplar wood, and treatment with exogenous ABA can mimic the wood anatomical features seen under drought (Yu et al., 2021). Recent application of genomic approaches has provided some insights into specific regulatory pathways, including identification of AREB1 and NAC transcription factors affecting vessel size in response to ABA in Arabidopsis and poplar (Li et al., 2019; Yu et al., 2019; Ramachandran et al., 2021). Auxin transport and biosynthesis disturbances have been shown to alter vessel width, frequency and grouping resulting from changes in cell differentiation patterns (Tuominen et al., 1997; Junghans et al., 2004; Johnson et al., 2018). However, less is known about how developing vessels perceive signals that influence their differentiation and size, how turgor and cell wall extensibility are regulated, or whether models of cell size regulation proposed for other organisms extend to trees.

We previously established a poplar population for functional genomics based on gene dosage (Henry et al., 2015; Bastiaanse et al., 2019; Bastiaanse et al., 2021). A large population of *Populus deltoides* x *P. nigra* hybrids was created, following gamma irradiation of pollen to create chromosomal deletions and additions (indels) in the resulting F1 progeny (Henry et al., 2015). The full genome of each F1 line was sequenced to precisely locate the chromosomal positions of deletions and insertions (Henry et al., 2015). Systems genomics approaches have been used in this population to dissect complex traits, including descriptions of genetic architecture and dosage quantitative trait loci (dQTL) that affect phenotype trait values when relative gene dosage is modulated, as well as putative mechanisms and individual candidate genes affecting traits (Bastiaanse et al., 2019; Bastiaanse et al., 2021). In previous work we used this unique population to determine heritabilities and identify dQTL affecting multiple vessel traits (Rodriguez-Zaccaro et al., 2021). The traits examined included mean vessel diameter (MVD), vessel frequency (VF), vessel grouping index (VGI), mean vessel circularity (MVC) and non-lumen fraction (NF; percentage of wood not comprised of vessel lumens), all of which showed significant heritabilities and behaved as classical quantitative traits influenced by many genes with modest individual effects.

Here we extend our previous study through analyses integrating transcript abundance data from wood-forming tissues with phenotypic and gene function data. Specifically, we use a systems genetics approach integrating information within a gene co-expression framework to identify putative genes and mechanisms underlying vessel traits. We discuss and test evidence for mechanisms affecting traits including stress perception and long-distance signaling, turgor regulation, cell wall extensibility, regulation of cell division and posttranslational modifications.

## Materials and methods

### Plant materials

A subset of 33 poplar F1 hybrid genotypes was selected from a larger group of 201 lines included in a previous study (Rodriguez-Zaccaro et al., 2021). Eleven genotypes were selected based on the presence of indel mutations spanning contiguous dQTL regions associated with both cMVD and cVF in chromosome 9. Six genotypes were selected based on the presence of indels spanning adjoining dQTL regions associated to cMVD in chromosome 16. Nine more genotypes were selected for their extreme cMVD and cVF phenotypes, regardless of indel mutation locations. Lastly, 7 genotypes were randomly selected as non-lesion controls.

Cuttings of the 201 selected genotypes were planted in individual 3-L pots inside a greenhouse and were established during a 2-month growth period. Three clonal replicates per line were then selected to include in a randomized complete block design across three benches. Trees were watered using a drip irrigation system and grown at 23°C. All trees were harvested for woody stem segments and coppiced in the late fall of 2018. These stems were processed to obtain stem and wood anatomical trait data as described previously (Rodriguez-Zaccaro et al., 2021). Trees were then allowed to regrow a main stem from the original cutting during the spring and early summer of 2019 under the same greenhouse conditions established in the previous growth season for sampling wood forming tissues for RNA extraction as described below.

### RNA-seq

The stems of 33 genotypes (2-3 replicates per genotype) were harvested in July of 2019. The final height before harvest was obtained for each of these trees, measuring from the point of emergence of the current year stem to the shoot apex. Stems were then harvested 5 to 10 cm from the point of emergence from the original cutting. Wood forming tissues were collected from harvested stems by gently peeling off the bark and scraping the uncovered tissue with double-edged razorblades. Tissues from each stem were placed in individual aluminum foil envelopes and quickly submerged in liquid nitrogen before being stored at -80°C.

Frozen tissue samples were ground to a fine powder using ceramic mortar and pestles containing liquid nitrogen. Total RNA was extracted from each sample and then purified through a protocol adapted from the TRIzol reagent user guide (Life Technologies) and the RNeasy handbook (QIAGEN). RNA samples were assessed for quantity and quality using a Qubit fluorometer (Invitrogen) and a Bioanalyzer (Agilent Technologies), respectively. A 3’ Tag-Seq mRNA library preparation kit (Lexogen QuantSeq) was used to construct cDNA libraries for each sample. Ninety-one libraries were multiplexed across three flow cell lanes of an Illumina HiSeq 4000 run generating 90 bp single end reads.

### Preprocessing and read counts table generation

FASTQ files were processed by removing unique molecular identifier (UMI) barcodes from sequences and adding them to sequence read names using the “extract” command in the “UMI-Tools” (v1.1.2) Python package (Smith et al., 2017). Files were then demultiplexed using 6 nucleotide long single indices through the Python tool “Allprep” (scripts available at: https://github.com/Comai-Lab/allprep). Reads were mapped to the *Populus trichocarpa* reference genome (v3.1) using the STAR software (v2.6) (Dobin and Gingeras, 2015). The resulting SAM files were then sorted using the package “SAMtools” (Li et al., 2009) and deduplicated to correct for amplification bias using UMIs in the sequence read names through the adjacency method of the “dedup” command in “UMI-Tools” (Smith et al., 2017). The “htseq-count” Python script (from the HTSeq package v0.13.5) was used to calculate the number of read counts mapped to each gene in the deduplicated SAM files (Anders et al., 2015). Normalization factors were calculated using the “calcNormFactors” function (TMM method) in the R package “edgeR” (v2.7) to correct for raw library sizes (Robinson et al., 2010). Genes with a maximum expression of less than 10 counts across all samples were filtered out as low-expressed or unexpressed genes. The “voom” function in the R package “limma” was used to transform counts into log2 CPM by implementing the previously calculated normalization factors. This function was also used to obtain weights to correct for differences in expression variance across genes (Law et al., 2014; Liu et al., 2015). A linear model using weighted least squares for each gene was then fitted using the “lmFit” function in “limma” (Ritchie et al., 2015). Model coefficients were extracted to produce a final gene expression dataset.

### Pearson correlations among traits

Trait data obtained from trees harvested in 2018 (Rodriguez-Zaccaro et al., 2021) were used to find correlations between all trait combinations within the 33-genotype subset. Pearson correlations and their corresponding P values were obtained using base R (v4.1.0), with significant correlations declared for P values of less than 0.05. Trait data include tree height at harvest (TH), mean vessel diameter (MVD), height-corrected mean vessel diameter (cMVD), vessel frequency (VF), height-corrected vessel frequency (cVF), vessel grouping index (VGI), mean vessel circularity (MVC), non-lumen fraction (NF), and bark thickness (BT).

### Weighted gene correlation network analysis

To identify candidate genes related to stem and wood anatomical traits, gene co-expression networks were constructed using the weighted gene correlation network analysis R package (WGCNA, v1.70-3; Langfelder and Horvath, 2008). All 33 sampled genotypes were included in the analysis, with 2-3 replicates per genotype. Twenty-six of these lines had large-scale indels throughout their genome, including 17 selected for the presence of indels in specific areas of chromosomes 9 and 16. A previous WGCNA study involving 164 dosage mutant lines from this pedigree found that half of all gene modules were localized within individual chromosomes, suggesting the creation of artificial indel-induced modules (Bastiaanse et al., 2021). To account for this effect, TMM and variance normalized log2 CPM gene expression data (described above) were processed into an indel-normalized data set. In indel lines, the expression of genes located within indel regions was replaced with the average expression of these genes in all other lines, eliminating the cis-effects of indels on expression (Bastiaanse et al., 2021). However, this approach can potentially compromise the ability to detect real biological correlations. Gene network construction and downstream analyses were thus run using both indel-normalized and non indel-normalized datasets to determine if normalization is necessary.

Co-expression networks were built by obtaining Pearson’s correlation coefficients for every expressed gene pair combination across all samples. Weighted adjacencies were then calculated through a transformation of coefficients involving a soft thresholding power of 12. This soft thresholding power was chosen for both networks to produce an 82% model fit to a scale-free topology. Topological overlap matrices (TOM) produced from the adjacencies were then used to calculate measures of topological overlap dissimilarities. Color-labeled modules of highly coexpressed genes were identified through the hierarchical clustering of these dissimilarities using a dynamic tree cutting height of 0.99. Eigengenes, or the weighted average of the expression of all genes within a module, were calculated and used to merge modules showing correlations of 0.75 or higher. Module stability was assessed for both networks through repeated resampling of 63% of all libraries, producing 49 co-expression network iterations. The physical locations of all co-expression modules across the Poplar genome were visualized using the R package “chromoMap” v0.3.1 (Anand and Rodriguez Lopez, 2022).

Stem and wood anatomical traits were tested for significant Pearson correlations with module eigengene values (P<0.05). Specifically, trait data obtained from trees harvested in 2018 for our previous study (Rodriguez-Zaccaro et al., 2021) were used to test for correlations with the gene expression of trees harvested in 2019 belonging to the same 33 genotypes. Two tree height datasets were included in the analysis, tree heights collected from 2018 plants, and tree heights collected from the 2019 plants harvested for RNA.

Gene co-expression modules were examined for functional enrichment through a GO enrichment analysis using *Arabidopsis thaliana* best BLAST hits of *P. trichocarpa* genes. The “treeGO” R package was used to perform hypergeometric tests to identify GO terms that are significantly overrepresented (P<0.05) within each module. Gene significance (GS) vs. module membership (MM) plots were obtained for biologically interesting modules. Gene significance is the correlation between the expression of a particular gene and a trait of interest, while module membership is the correlation between the expression of a gene and the eigengene value of the module.

### Dosage response analysis

We tested for the presence of dosage responsive genes within two previously identified cMVD and cVF dQTL regions (Rodriguez-Zaccaro et al., 2021). One dQTL region is located on chromosome 9 (from 6.3 to 6.8 Mbp) and was highly correlated to both cMVD and cVF. The other dQTL region was located on chromosome 16 (from 0 to 0.5 Mbp) and was highly correlated to cMVD. Genotypes were grouped into relative dosage score (RDS) categories determined by the presence of insertions or deletions at each dQTL region. Lines with deletions have an RDS of 0.5, lines without indels have an RDS of 1.0 and lines with insertions have an RDS of 1.5. All genes expressed within a dQTL region were then tested for significant differences in gene expression across RDS categories through ANOVAs (P<0.05). Three lines with deletions, 4 lines without lesions and 2 lines with insertions covering the chromosome 9 region were included in an ANOVA. Four lines with deletions, 4 lines without lesions and 2 lines with insertions spanning the chromosome 16 region were included in a separate ANOVA. The 4 non-lesion lines were randomly selected. Each analysis was followed by a Tukey’s honest significance *post hoc* test. Analyses were performed using base R (v4.1.0).

## Results

Genotypes in the study were selected either to provide indel coverage of dQTL identified in our previous study (Rodriguez-Zaccaro et al., 2021) or to include genotypes with extreme trait values as well as control genotypes without indels (Materials and Methods). Phenotypic traits measured for each clone are summarized in **Table 1** and include wood anatomical and cell morphological traits relevant to stem hydraulic properties measured in our previous study (Rodriguez-Zaccaro et al., 2021). Pearson correlation coefficients were calculated for all stem and wood anatomical trait combinations across the 33 hybrid poplar genotypes (**Supplementary Figure 1**). Tree height was positively correlated to both mean vessel diameter (R=0.66, P<0.01) and bark thickness (R=0.70, P<0.001), and strongly negatively correlated to vessel frequency (R=-0.85, P<0.001). Vessel frequency in turn showed a strong negative correlation to mean vessel diameter (R=-0.82, P<0.001) and bark thickness (R=-67, P<0.001). Height corrected mean vessel diameter and height corrected vessel frequency were negatively related (R=-0.67, P<0.05). Vessel grouping index, mean vessel circularity and non-lumen fraction showed no significant correlations to any trait.

**Table 1:** Stem and wood anatomical traits examined across 33.

Trait	Abbr.	Units
Tree height	TH	cm
Vessel frequency	VF	vessels/mm^2^ xylem
Height-corrected VF	cVF	unitless
Mean vessel diameter	MVD	µm
Height-corrected mean vessel diameter	cMVD	unitless
Mean vessel circularity	MVC	unitless
Vessel grouping index	VGI	vessels/vessel group
Non-lumen fraction	NF	unitless
Bark thickness	BT	µm

F1 hybrid poplar genotypes (Populus deltoides × nigra).

### Constructing gene co-expression networks for wood forming tissues

**Figure 1:**
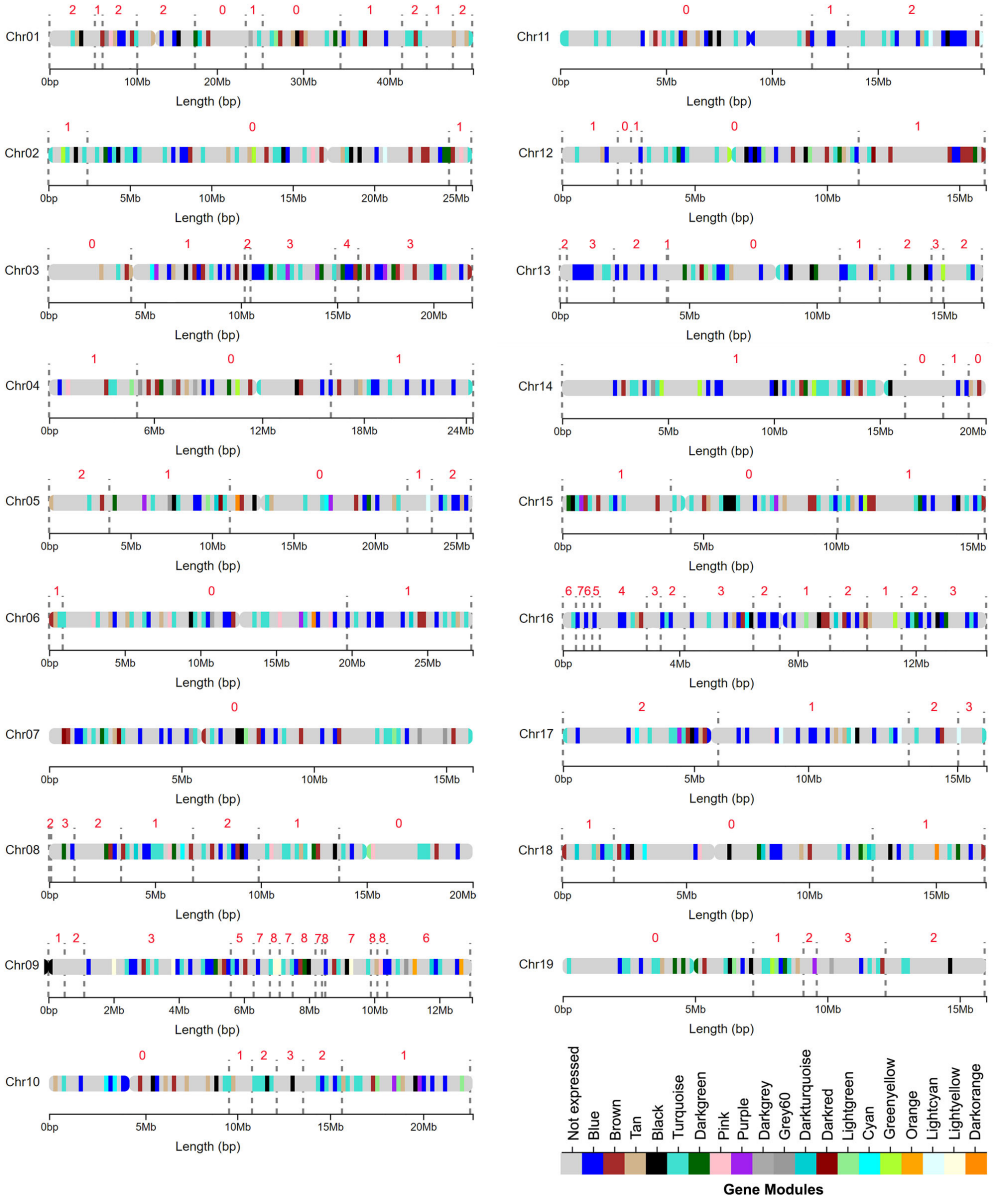
Broadly distributed genomic locations of gene modules identified through a weighted gene coexpression network analysis (WGCNA) using a non indel normalized dataset. Colored regions along chromosomes represent areas with a high density of genes in a particular module. The grey dotted lines mark the edges of indel regions. The number above each region represents the number of genotypes with indels in that region.

RNAseq read count data from wood forming tissues from each clone was used to construct gene co-expression networks to identify broad biological pathways and candidate genes related to stem and wood anatomical traits (Materials and Methods). Some genotypes included in the study have overlapping indel mutations that could potentially cause the creation gene co-expression modules reflecting dosage responsive genes localized to the overlapping indels, and these potential artifacts can be addressed using a normalization procedure (Baastianse et al., 2020). To determine if normalization was warranted for data in the current study, co-expression networks were constructed and compared for both non indel-normalized and indel-normalized gene expression datasets (Materials and Methods). Nineteen modules consisting of highly coexpressed genes were identified within both networks (**Supplementary Figure 2**). Module sizes were similar across non-normalized and indel normalized networks and ranged from 46 to 4711 genes per module (**Supplementary Table 1**). Hypergeometric tests showed a highly significant overrepresentation of genes of the non-normalized modules in each normalized module (P<0.00001) consistent with similar networks being recovered by the two approaches. A module stability analysis showed that individual modules within each network persist across many network construction subsampling iterations, suggesting that the modules are robust within both networks (**Supplementary Figure 3**). Similar to results using indel-normalized data (**Supplementary Figure 4**), genes from non indel-normalized modules were widespread throughout the genome, with genes from every module represented across most chromosomes (**Figure 1**). The light-yellow and orange modules were the least widely distributed, with most of their genes located on chromosome 9 (**Supplementary Figure 5**), and correlations of genes within these modules and traits should thus be evaluated with caution. However, regions in chromosome 9 with high indel coverage depth also included many genes that were assigned to other modules (**Supplementary Figure 6**), and there was no enrichment of genes from any particular module for a 0.5Mbp bin on chromosome 16 spanned by indels (**Supplementary Figure 7**). Together these results did not support the need for global data normalization. Because normalization changes expression values for genes in indel regions for genotypes that are most informative for examining dosage-trait relationships it would reduce the power to detect correlations among modules or genes and phenotypes, and thus results are presented using non-indel normalized networks for the remainder of analyses. However, transcript counts, annotations and module membership for individual genes from both the non-indel normalized and the indel normalized network are provided in **Supplementary Tables 2**, **3**, respectively.

### Gene modules correlate with phenotypic traits

**Figure 2:**
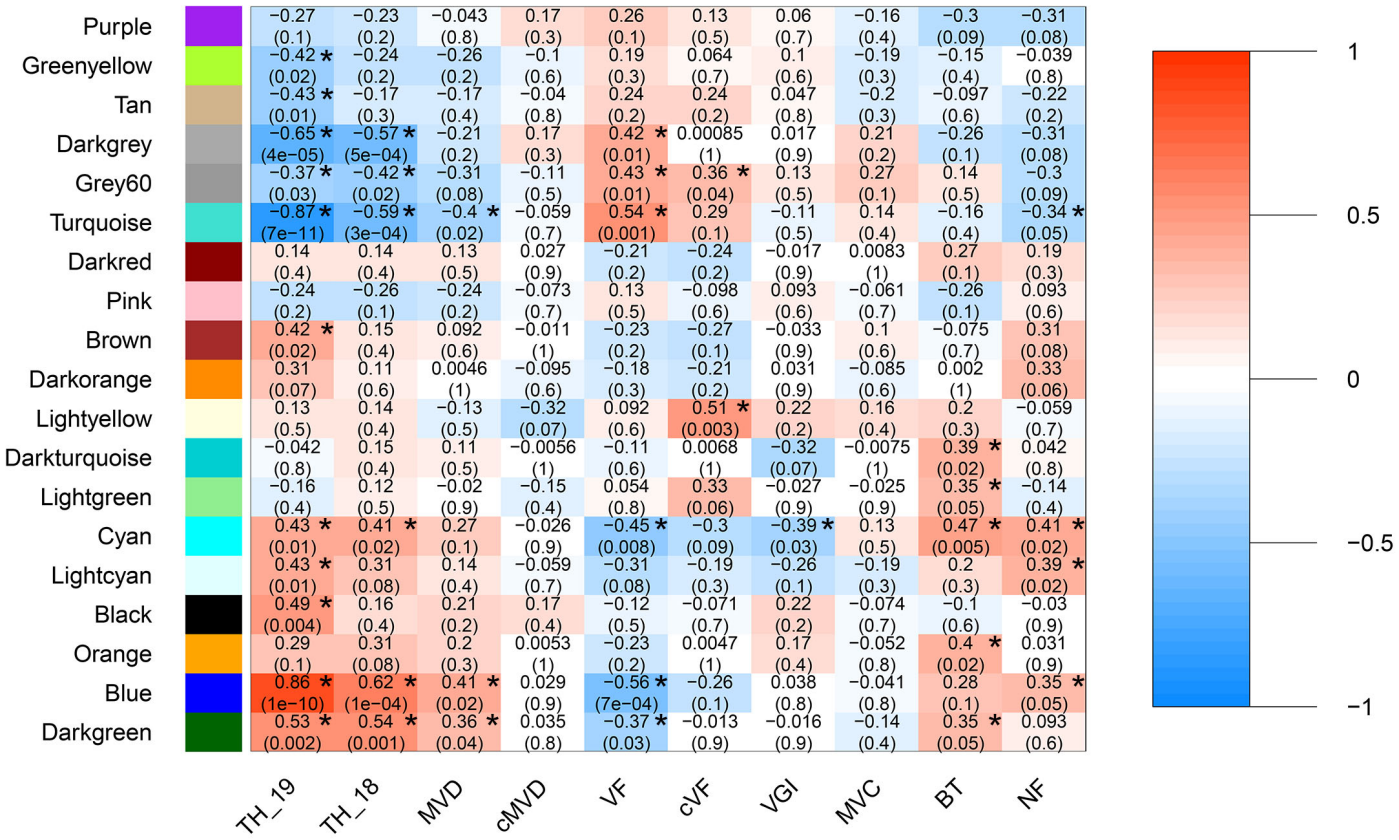
Module eigengene and trait correlations across 33 hybrid poplar genotypes. Module eigengene and trait correlations across 33 hybrid poplar genotypes. Wood anatomical traits include mean vessel diameter (MVD), height-corrected mean vessel diameter (cMVD), vessel frequency (VF), height-corrected vessel frequency (cVF), vessel grouping index (VGI), mean vessel circularity (MVC), and non-lumen fraction (NF). Stem traits include tree height (TH) and bark thickness (BT). Each box includes a Pearson’s correlation coefficient represented by a color bar (color scale on right shows ranged of positive and negative correlations) and a P value. P values ≤ 0.05 are marked with an asterisk.

Correlations were calculated between the eigengene value of expression for each module and stem anatomical and vessel morphological trait data (P<0.05; **Figure 2**). Tree height in both years were the most strongly correlated to the darkgrey, grey60, turquoise, cyan, blue, and dark-green modules, which together comprised about 64% of all expressed genes, consistent with the complex polygenic nature of this trait. Modules tan, brown, lightcyan and black were only associated with tree height in the year transcript levels were measured, 2019. No modules were uniquely associated with tree height in 2018. Together these findings suggest that while overall transcript levels and module eigengene values associated with height were similar across the two years of the study, correlations were weaker when height and transcript abundance were not obtained in the same year. Thus, it is likely that the power to detect correlations between traits below (also measured in 2018) and modules is weakened by variation in plant growth between the two years.

All anatomical and vessel morphological traits were correlated with one or more modules, with the exception of height corrected mean vessel diameter and mean vessel circularity (P <0.05; **Figure 2**). Vessel frequency was the most highly correlated to any gene module across both networks and had the greatest number of significant correlations to modules (P <0.05; **Figure 2**). Vessel frequency had positive correlations to the darkgrey, grey60, and turquoise (R=0.42, 0.43 and 0.56, respectively) and negative correlation to the blue and darkgreen modules (R=-0.56 and -0.37). Similarly, mean vessel diameter was significantly correlated to the turquoise, blue and darkgreen modules (R=0.4, 0.41 and 0.36 respectively). Height corrected vessel frequency was positively related to the light-yellow (R=0.51) and the grey60 (R=0.36). Vessel grouping index was significantly related to the cyan module (R=-0.39), while non-lumen fraction was correlated to the turquoise (R=-0.34), cyan (R=0.41), light-cyan (R=0.39) and blue (R=0.35) modules. Bark thickness was positively correlated with the darkturquoise (R=0.39), lightgreen (R=0.35), cyan (R=0.47), orange(R=0.40) and blue (R=0.35) modules.

Modules associated with multiple traits can reflect common regulatory mechanisms shared across traits. Modules that correlated with vessel traits also correlated with tree height, with the exception of the unique correlation of the lightyellow module with height corrected vessel frequency. For example, three of the modules correlated with mean vessel diameter (turquoise, blue, orange) were also correlated with height and in the same direction. Similarly, all six of the modules correlated with vessel frequency were also correlated with height, but in the opposite direction. The positive correlation of height with mean vessel diameter trait and negative correlation with vessel frequency (**Supplementary Figure 1**) are thus reflected in these co-expression modules. Such correlations may indicate that a module’s biological function contributes to variation in vessel traits and indirectly influences height growth, for example by affecting water flow rates during growth, or that the module’s functions directly influence both sets of traits.

Mean vessel diameter and vessel frequency are negatively correlated traits (**Supplementary Table 1**), and all three modules significantly correlated with mean vessel diameter (turquoise, blue and darkgreen) were also correlated with vessel frequency, while three additional modules (darkgrey, grey60, and cyan) were unique to vessel frequency and could reflect mechanisms specific to this trait. One of the two modules correlated with height corrected vessel frequency was also correlated with vessel frequency (grey60), while the lightyellow module was unique to height corrected vessel frequency.

### Gene module GO enrichment analyses

**Figure 3:**
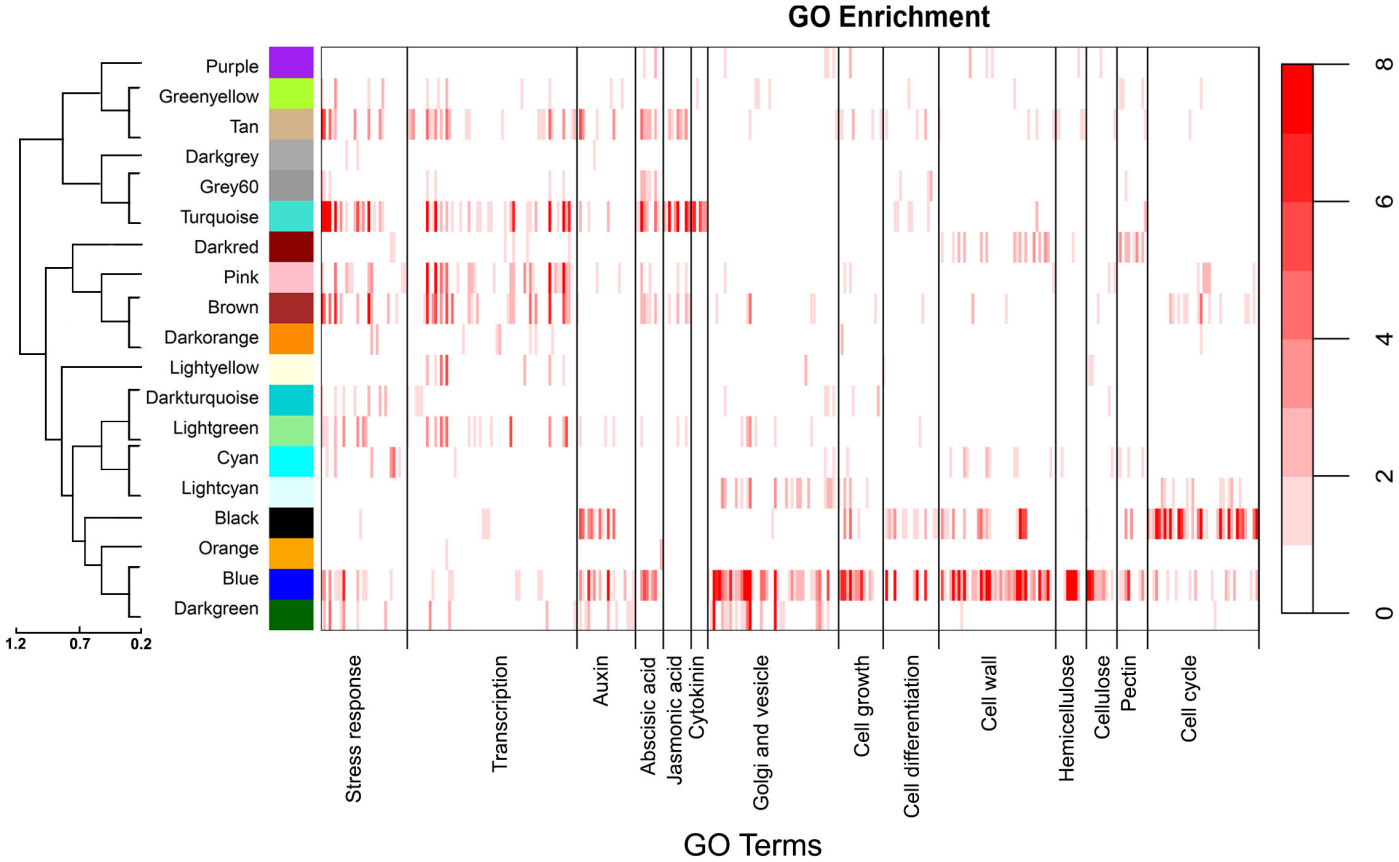
Enrichment analysis heatmaps showing significant overrepresentation (P<0.05) of GO terms within gene modules. The color bars represent the -log10 of P values (scale to right) generated from hypergeometric tests. Each major category indicated on the x axis is composed of individual child GO categories represented by individual bars. **Supplementary File 2** lists all child categories and statistical test values for each module. The dendrogram to the left shows the relationships among modules.

Co-expression modules were next assigned broad biological functions through GO enrichment analyses (Materials and Methods) to provide insights into potential mechanisms affecting traits. GO enrichment categories and statistics are provided in **Supplementary Table 4**. As shown in **Figure 3**, the turquoise module associated with height, mean vessel diameter and vessel frequency was significantly enriched in stress response, transcription regulation and hormone related terms, among others (P<0.05). Response to stress was one of the most significantly overrepresented terms, with 769 genes with this annotation included in the module. Response to cold (125 genes) and water deprivation (134 genes) were the most significantly overrepresented specific stress response terms. After stress response, transcription regulation related terms where among the most numerous and significantly overrepresented, with 446 genes in the turquoise module associated with nucleic acid-templated transcription. Lastly, this module was highly enriched in response to hormone terms, which together encompassed 330 genes. In particular, genes annotated with response to abscisic acid terms were the most numerous and overrepresented within this category, with 141 genes included in the turquoise module. Response to jasmonic acid and cytokinin related terms were also significantly overrepresented.

The blue module was also correlated with height, mean vessel diameter, and vessel frequency but in the opposite directions as the turquoise module. In contrast to the turquoise module, the blue module was significantly enriched in genes associated with vesicle transport, Golgi processes, cell wall, cell growth and cell differentiation related terms, among others (**Figure 3**). Vesicle mediated transport related terms were the most significantly overrepresented (193 genes), with 109 of these genes annotated with Golgi vesicle transport and organization terms. In addition, the blue module was highly enriched in cell wall organization and biogenesis terms, with 149 genes with these annotations included in the module. Hemicellulose was the most overrepresented among specific cell wall component related terms, followed by pectin and cellulose. Lastly, cell growth (133 genes) and differentiation (145 genes) associated terms were greatly overrepresented in the blue module. The blue module would thus be consistent with genes and mechanisms associated with cell differentiation and cell wall biosynthesis, a critical feature of vessel element differentiation. The darkgreen module was also correlated with height, mean vessel diameter, and vessel frequency (**Figure 2**) but in contrast to the turquoise and blue modules was not highly enriched in genes associated with abscisic acid (**Figure 3**).

The cyan module showed the strongest correlation to BT and the third strongest correlation to VF, after the turquoise and blue modules (**Figure 2**). This module was enriched in stress response and cell wall related annotations (**Figure 3**). The light-yellow and grey60 modules in the non indel-normalized network were correlated to height corrected vessel frequency and were enriched in transcription and stress response related terms, respectively (**Figure 3**).

### Gene significance vs module membership plots

**Figure 4:**
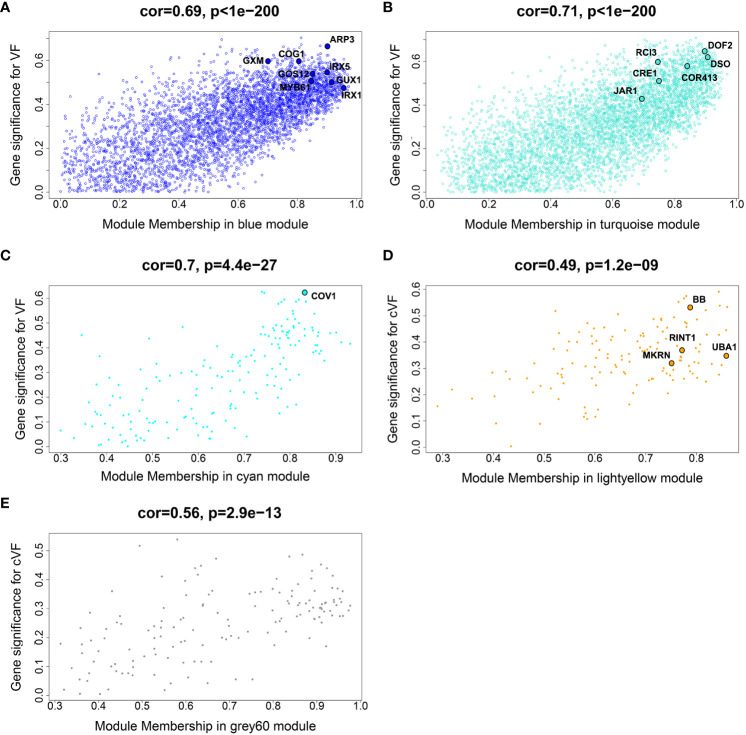
Gene significance (GS) vs module membership (MM) scatterplots of selected modules identified from a non indel normalized dataset. The Blue, Turquoise and Cyan module plots **(A-C)** show GS for vessel frequency (VF), while the Lightyellow and Grey60 plots **(D, E)** show GS for height-corrected vessel frequency (cVF). These modules showed highly significant correlations between GS and MM. Representative genes with high GS and MM are highlighted in the modules and included in **Table 2**.

Biologically relevant modules were next examined for candidate genes related to wood anatomical traits through gene significance (GS – a measure of correlation between a gene’s expression and a phenotype) vs module membership (MM – a measure of correlation between a gene’s expression and the eigengene of the module) plots (Langfelder and Horvath, 2008), as shown in **Figure 4**. While this approach can be used to dissect any module-trait combination, we focus on vessel frequency-related candidate genes, as this trait showed the strongest and most numerous correlations to modules among the examined wood anatomical traits, as well as height corrected vessel frequency candidate genes. Genes with the greatest GS and MM values within overrepresented GO categories were identified as candidate genes within each selected module (Langfelder and Horvath, 2008) and are listed in **Table 2**. Candidate genes reflect potential mechanisms affecting vessel traits, including stress response and signaling, Golgi and vesicle-related genes, cell wall -related genes, and cell differentiation-related genes (**Table 2**). Some of these candidate genes have been functionally characterized in other species and connected to directly or indirectly to vessel traits, such as the orthologs of Arabidopsis genes IRREGULAR XYLEM 1 (IRX1) and IRX5 that encode secondary cell wall-related cellulose synthases (Taylor et al., 2003). Loss of function mutations of these genes in Arabidopsis result in a “collapsed vessels” phenotype (Turner and Somerville, 1997). Interestingly, IRX1 mutants have also been shown to have enhanced drought and osmotic stress tolerance in Arabidopsis (Chen et al., 2005). Other notable candidates include an ortholog of Arabidopsis CONTINUOUS VASCULAR RING (COV1), which encodes a protein associated with Golgi morphology and vacuolar protein sorting (Shirakawa et al., 2014). Arabidopsis cov1 mutants are characterized by extension of vascular bundles boundaries, suggesting COV1 is a negative regulator of vascular differentiation in stems (Parker et al., 2003). An ortholog of VASCULAR-RELATED NAC DOMAIN 2 (VND2) encodes a transcription factor that, in Arabidopsis, has been shown to modify respond to ABA during stress to modify the differentiation rates of vessel elements (Ramachandran et al., 2021). Lastly an ortholog of BIG BROTHER (BB) was identified, which in Arabidopsis encodes a RING-finger E3 ubiquitin ligase that represses organ growth, potentially by degrading growth-stimulating proteins (Disch et al., 2006). Interestingly Arabidopsis BB is dosage sensitive (Disch et al., 2006) and has been hypothesized to uncouple cell proliferation from cell elongation (Cattaneo and Hardtke, 2017).

**Table 2:** Candidate genes related to vessel frequency (VF) and height corrected vessel frequency (cVF) across four gene modules.

Gene	Module	Trait	GS	MM	Symbol	GO Terms (BP)
Potri.007G122100	turquoise	VF	0.60	0.76	RCI3	Response. to cold, desiccation, oxidative stress
Potri.004G149100	turquoise	VF	0.58	0.84	COR413	Response to water deprivation
Potri.002G013200	turquoise	VF	0.52	0.90	ERD10	Response to cold, water deprivation
Potri.014G104200	turquoise	VF	0.50	0.78	CIPK9	Response to cold, salt stress
Potri.012G044300	turquoise	VF	0.48	0.95	NUDT4	Response to water deprivation, salt stress
Potri.004G056900	turquoise	VF	0.64	0.91	DOF2	Regulation of transcription
Potri.012G100200	turquoise	VF	0.60	0.79	CAL	Regulation of transcription
Potri.005G073100	turquoise	VF	0.63	0.92	DSO	Response to abscisic acid
Potri.014G030700	turquoise	VF	0.59	0.93	CDPK1	ABA activated signaling pathway
Potri.014G095500	turquoise	VF	0.42	0.69	JAR1	JA metabolic process
Potri.010G108200	turquoise	VF	0.32	0.80	JAI3	JA metabolic process
Potri.010G102900	turquoise	VF	0.52	0.77	CRE1	CK activated signaling pathway
Potri.003G136300	turquoise	VF	0.50	0.80	CRF2	CK activated signaling pathway
Potri.010G177200	blue	VF	-0.60	0.82	COG1	Golgi vesicle-mediated transport
Potri.014G066800	blue	VF	-0.54	0.84	GOS12	Golgi vesicle-mediated transport
Potri.018G086600	blue	VF	-0.50	0.85	GC1	Golgi organization
Potri.019G076300	blue	VF	-0.59	0.73	GXM	Xylan metabolic process
Potri.013G102200	blue	VF	-0.55	0.80	GXM	Xylan metabolic process
Potri.007G107200	blue	VF	-0.51	0.95	GUX1	Xylan biosynthetic process
Potri.002G257900	blue	VF	-0.54	0.91	IRX5	Cellulose biosynthetic process
Potri.011G069600	blue	VF	-0.48	0.96	IRX1	Cellulose biosynthetic process
Potri.018G062700	blue	VF	-0.53	0.95	Pectin lyase	Carbohydrate metabolic process
Potri.008G182700	blue	VF	-0.67	0.90	ARP3	Cell growth
Potri.014G115000	blue	VF	-0.50	0.75	XET33	Cell growth
Potri.013G001000	blue	VF	-0.52	0.84	MYB61	Cell differentiation
Potri.012G126500	blue	VF	-0.45	0.78	VND4	Xylem vessel differentiation
Potri.005G148000	cyan	VF	-0.63	0.83	COV1	Stem vascular tissue pattern formation
Potri.009G062100	lightyellow	cVF	0.53	0.79	BB	Negative regulation of cell proliferation
Potri.009G020600	lightyellow	cVF	0.37	0.77	RINT1	Cell cycle progression
Potri.009G075700	lightyellow	cVF	0.35	0.86	UBA1	Cell aging
Potri.009G062600	lightyellow	cVF	0.32	0.75	MKRN	Cell aging

Gene modules were selected based on significant correlations to VF and cVF and GO enrichment analysis results. Candidate genes with the greatest module membership (MM) and gene significance (GS) within overrepresented GO categories were selected from each module. *P. trichocarpa* genome v3.1.

### Dosage responsiveness further narrows candidate genes underlying dQTL

**Figure 5:**
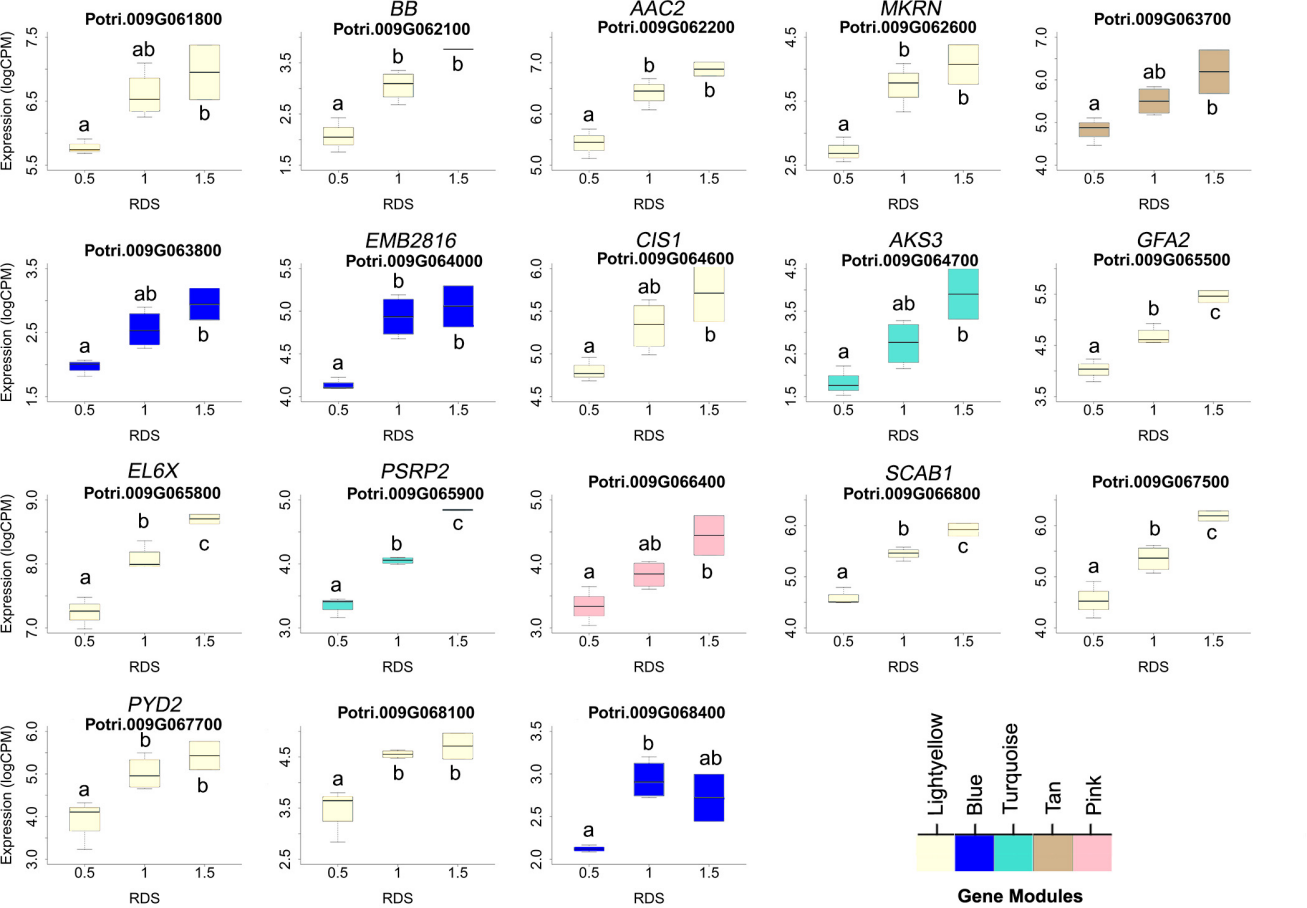
Putative dosage-responsive genes within a previously identified dosage dependent QTL (dQTL) in chromosome 9. Each boxplot shows gene expression for genotypes grouped by relative dosage score (RDS) at each gene locus within the chromosome 9 dQTL region (6.3-6.8 Mbp). Only genes that had significantly different expression levels across RDS categories were included (determined by ANOVAs). Tukey pairwise comparisons were used to show significant differences in gene expression values between RDS groups; means that share a letter are not significantly different (p > 0.05). Module membership for each gene is indicated by colors (see scale for names).

**Figure 6:**
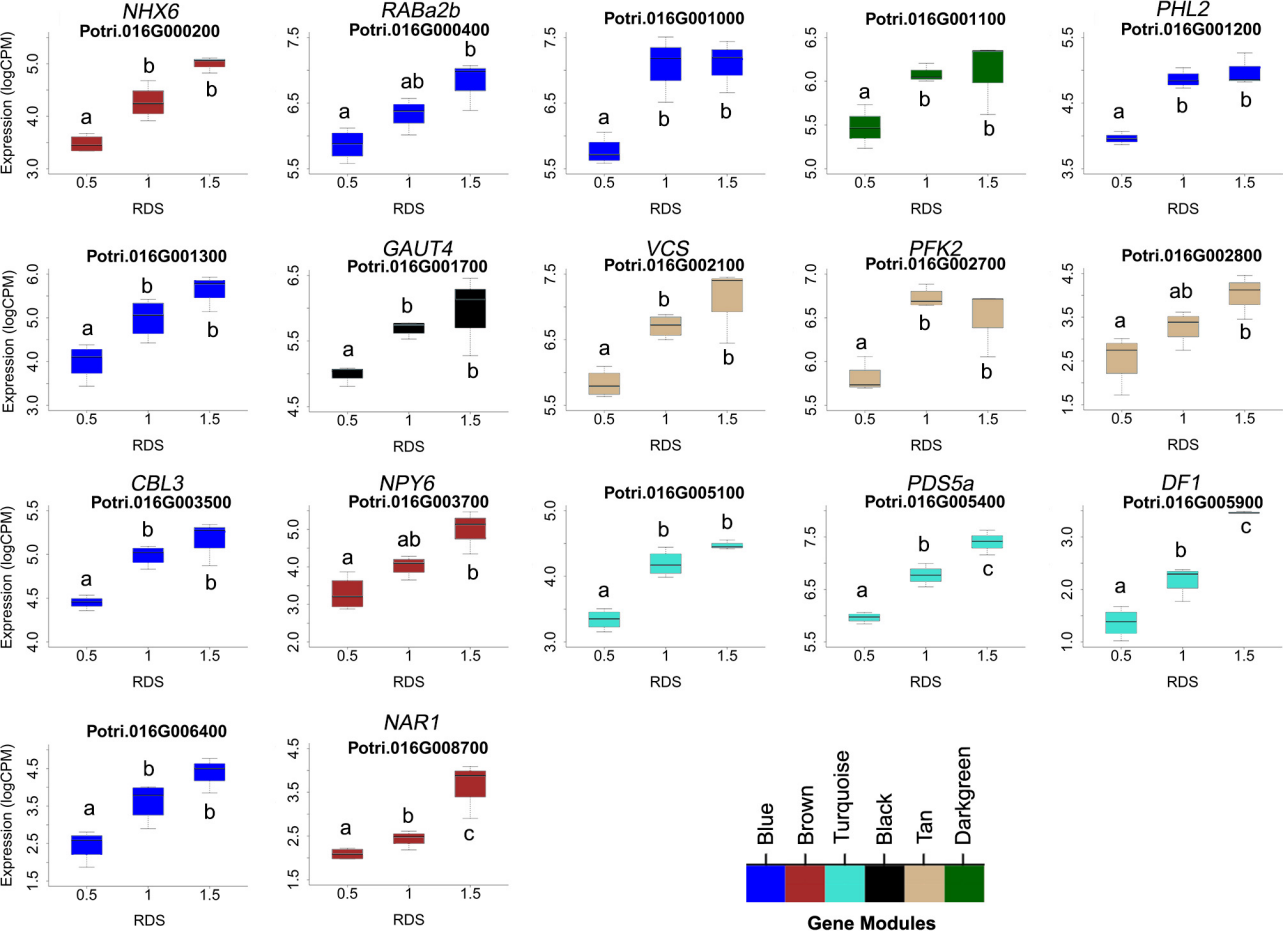
Putative dosage-responsive genes within a previously identified dosage dependent QTL (dQTL) in chromosome 16. Each boxplot shows gene expression for genotypes grouped by relative dosage score (RDS) at each gene locus within the chromosome 16 dQTL region (0-0.5 Mbp). Only genes that had significantly different expression levels across RDS categories were included (determined by ANOVAs). Tukey pairwise comparisons were used to show significant differences in gene expression values between RDS groups; means that share a letter are not significantly different (p > 0.05). Module membership for each gene is indicated by colors (see scale for names).

One prediction is that the causative gene(s) underlying vessel trait dQTL should be expressed in wood forming tissues and should themselves respond to dosage in terms of expression. We tested genes within dQTL regions on chromosome 9 (from 6.3 to 6.8 Mbp) associated with height corrected mean vessel diameter and height corrected vessel frequency and chromosome 16 (from 0 to 0.5 Mbp) associated with height corrected mean vessel diameter (Rodriguez-Zaccaro et al., 2021) for genes whose expression changes within relative dosage. Lines were placed in relative dosage score (RDS) groups determined by the presence of insertions or deletions spanning each dQTL region. Forty-two genes were expressed out of a total of 75 annotated genes within the chromosome 9 region. Eighteen of these genes showed significantly different expression across lines with different RDS values, suggesting dosage responsiveness (P<0.05). The expression of most of the 18 genes showed a positive relationship to their dosage in the region, with more copies of a gene leading to greater expression (**Figure 5**). Slopes obtained from linear regressions of gene expression data across different RDS lines ranged from 0.70 to 2.04, showing a wide variation in the effect of dosage on expression (**Table 3**). None of the genes within this group scored high on gene significance/module membership plots, however (**Figure 4**). Within the chromosome 16 region, 46 out of 103 annotated genes were expressed in wood forming tissues. Seventeen of these genes showed significantly different expression across different RDS line categories (P<0.05; **Figure 6**). As expected, the expression of these genes was positively related to their gene dosage, with lines with greater RDS values showing greater expression (**Figure 6**). Slopes obtained from linear regressions of gene expression data across RDS categories ranged from 0.83 to 2.12, showing a large variation in the effect of dosage on expression (**Table 4**). Within this group, two genes scored high on gene significance/module membership plots (**Table 4**), Potri.009G062100 (ortholog of BIG BROTHER ubiquitin E3 ligase) and Potri.009G062600 (ortholog of a MAKORIN-like ubiquitin E3 ligase).

**Table 3:** Putative dosage-responsive genes within a previously identified dosage dependent QTL (dQTL) region in chromosome 9 (6.3-6.8 Mbp).

Gene	P	Slope	Symbol	GO Term (BP)
Potri.009G061800	2.4E-2	1.22	DIS3	Nucleic acid bond hydrolysis
Potri.009G062100	1.6E-3	1.71	BB	Regulation of cell proliferation
Potri.009G062200	1.7E-3	1.50	AAC2	Transmembrane transport
Potri.009G062600	5.0E-3	1.41	MKRN	Protein ubiquitination
Potri.009G063700	3.1E-2	1.37	Transmembrane	–
Potri.009G063800	1.6E-2	1.00	RNA-binding	mRNA processing
Potri.009G064000	5.6E-3	0.98	LSM7	Nuclear mRNA degradation
Potri.009G064600	3.6E-2	0.92	SWAP	RNA splicing
Potri.009G064700	1.7E-2	2.04	Basic HLH	Regulation of transcription
Potri.009G065500	5.0E-4	1.42	GFA2	Response to heat
Potri.009G065800	5.7E-4	1.48	Ribosomal L6	Translation
Potri.009G065900	8.5E-6	1.50	RNA-binding	Nucleic acid binding
Potri.009G066400	1.7E-2	1.09	Trans. initiation	Translation initiation
Potri.009G066800	1.3E-4	1.36	SCAB1	Actin filament organization
Potri.009G067500	1.8E-3	1.64	RNA-binding	mRNA splicing
Potri.009G067700	2.2E-2	1.61	PYD2	Pyrimidine catabolic process
Potri.009G068100	7.3E-3	1.37	SKIP2	Protein ubiquitination
Potri.009G068400	9.9E-3	0.70	Methyltrans.	Protein methylation

Genotypes were grouped by RDS at each gene locus within the dQTL region. An ANOVA was performed using gene expression data across different RDS groups. Only genes that had significantly different expression levels across RDS categories were included (P<0.05). The slope obtained from the linear regression of gene expression data across RDS groups is included to show the magnitude of the effect of dosage on expression.

**Table 4:** Putative dosage-responsive genes within a previously identified dosage dependent QTL (dQTL) region in chromosome 16 (0-0.5 Mbp).

Gene	P	Slope	Symbol	GO Term (BP)
Potri.016G000200	2.5E-3	1.55	NHX6	Transmembrane transport
Potri.016G000400	1.9E-2	0.94	RABA2b	GTP binding
Potri.016G001000	2.9E-3	1.76	Alpha beta-hydrolase	Hydrolase activity
Potri.016G001100	3.3E-3	0.86	PHL7	Regulation of transcript.
Potri.016G001200	4.4E-5	1.30	PHL2	Phosphate starvation resp.
Potri.016G001300	1.6E-2	1.78	Cl- channel regulator	Zinc ion binding
Potri.016G001700	2.2E-4	1.17	GAUT4	Glycosyltransferase
Potri.016G002100	7.5E-4	1.44	Autoant. RCD8	mRNA binding
Potri.016G002700	2.8E-4	1.08	PFK2	Glycolytic process
Potri.016G002800	4.4E-2	1.59	O-Glycosyl hydrolase	Carbohydrate metabolism
Potri.016G003500	7.4E-4	0.83	CBL3	Detection of calcium ion
Potri.016G003700	1.2E-2	1.64	NPH3	Protein ubiquitination
Potri.016G005100	6.9E-4	1.31	Sec14p	–
Potri.016G005400	1.5E-4	1.47	Sister chro. cohesion	–
Potri.016G005900	1.3E-3	1.91	DNA binding	–
Potri.016G006400	1.1E-2	2.13	Protein kinase	Cellular resp. to nitrogen
Potri.016G008700	1.6E-4	1.43	Ferredo. hydrogenase	–

Genotypes were grouped by RDS at each gene locus within the dQTL region. An ANOVA was performed using gene expression data across different RDS groups. Only genes that had significantly different expression levels across RDS categories were included (P<0.05). The slope obtained from the linear regression of gene expression data across RDS groups is included to show the magnitude of the effect of dosage on expression. *P. trichocarpa* genome v3.1.

## Discussion

In this study we used a unique poplar germplasm resource (Henry et al., 2015) to identify potential mechanisms and individual genes influencing vessel traits. The poplar pedigree utilized carries dosage variation from genomically defined insertions and deletions and is an unusually rich source of genetic and phenotypic variation for dissecting complex traits, as shown here and in previous studies (Bastiaanse et al., 2019; Bastiaanse et al., 2021; Rodriguez-Zaccaro et al., 2021). We used a gene co-expression approach that allowed integration of RNAseq transcript abundance profiling data with vessel traits and other data types across clonally replicated genotypes to identify potential mechanisms and candidate genes correlated with vessel traits. Below, we discuss the results from the study against the backdrop of known and anticipated mechanistic components of vessel traits.

To fully understand vessel traits, a minimal list of component traits that must be explained include 1) perception of water stress and long distance signaling across organs and tissues, 2) cell fate decisions of cambial daughter cells that ultimately determine vessel patterning, 3) regulation of vessel morphology and diameter. Our gene expression data from wood forming tissues is likely not ideal for identifying genes and mechanisms related to water/osmotic stress perception, and it is not known to what degree osmotic stress is directly perceived in wood forming tissues or responds to signals originating in roots. However, at the level of mechanisms our data reflected a role for ABA in both mean vessel diameter and vessel frequency (see turquoise module **Figures 2**, **3**). ABA has been previously implicated as a primary drought stress signal whose manipulation can change drought response (Yu et al., 2019) and vessel trait phenotypes in poplar (Popko et al., 2010). This same gene module was also enriched for stress response genes as well as other hormone related genes, including jasmonic acid, which has been shown to interact with ABA in other plant systems during stress (de Ollas et al., 2015), and auxin which has also been shown to modify vessel patterning and size (Junghans et al., 2004; Johnson et al., 2018).

The sequence of events in vessel element differentiation includes specification of vessel element cell fate, cessation of cell division and initiation of differentiation, cell expansion, and secondary cell wall synthesis prior to programmed cell death (Turner et al., 2007). We were unable to identify mechanisms or candidate genes that are unique to the process by which cambial daughter cells are specified to differentiate as vessels and thus determine vessel patterning traits (vessel frequency, vessel grouping index) from our results. Unfortunately, our experimental design was not able disentangle how vessel patterning versus size could be uniquely regulated, which would have required experimental treatments capable of breaking the observed phenotypic correlations between these traits. Thus, our experimental design likely resulted in some gene module-trait correlations that indirectly reflect trait-trait correlations. It is also possible that at least at some early stage of cell specification and differentiation there are common regulatory mechanisms affecting both traits. Although purely speculative, a general type of mechanism that could connect vessel patterning and size could be envisioned where a cell specified as a vessel represses expansion of neighboring vessels, for example by localized diffusion of a repressive signal.

Cell expansion and final cell size reflect the interplay of turgor pressure and resistance of the cell wall (Ali et al., 2023). Clearly turgor pressure is a central aspect of vessel size, as illustrated by the massive expansion of vessel elements in contrast to the limited expansion of neighboring cells that differentiate as fibers that also synthesize secondary cell walls. We did not find clear evidence for enrichment of genes associated with turgor-related process at the level of GO analysis, which could reflect that this process is not globally regulated at the level of transcription. In support of that notion, recent results in Arabidopsis identified posttranslational regulatory mechanisms affecting turgor-driven cell expansion (Chaudhary et al., 2023). Specifically, the Arabidopsis receptor kinase FERONIA (FER) was implicated in regulating turgor-driven cell expansion in response to brassinosteroids, and FER is postranslationally regulated through phosphorylation by the BRASSINSTEROID-INSENSITIVE 2 kinase (Chaudhary et al., 2023). However, we did find strong signatures of cell wall biosynthesis and modification-related genes associated with both vessel frequency and diameter (**Figure 3**). One possibility is cell size is affected in part by the timing of biosynthesis or changes in mechanical properties in cell walls that affect the resistance of the cell wall to turgor during expansion, with lignification of the rigid secondary wall ultimately preventing further cell expansion. While many secondary cell wall-related mutants in plants have not been specifically examined for vessel element patterning or diameter phenotypes, there are examples of wall-related genes and mutants with altered vessel morphologies. For example, an ABA responsive AREB1 transcription factor in poplar regulates both the diameter and frequency of vessels. AREB1 was also shown to regulate poplar NAC transcription factors (Li et al., 2019), which have been shown to include master regulators capable of initiating vessel-like differentiation and cell wall biosynthesis in other cell types when overexpressed (Kubo et al., 2005; Yamaguchi et al., 2010). That both size and frequency are affected in AREB1 misregulated poplars may indicate that specification of vessel cell identity is a key point of regulation (Li et al., 2019). However, the apparent lack of significant cell expansion in other cell types induced to differentiate as vessel by ectopic expression of NACs (e.g. Yamaguchi et al., 2010) may indicate that cell identity including cell expansion is specified upstream or in parallel of NAC cell wall regulation.

The regulation of cell size is a fundamental issue for plant development but remains poorly understood. The determination of vessel element diameter is especially complex because it is highly responsive to environmental conditions. Three generalized models of cell size regulation have been proposed based on findings in the more simplified yeast system: the “timer”, “sizer” and “adder” models (D’Ario and Sablowski, 2019), all of which evoke a key role for cell division in determining final size. The timer model predicts that cells divide at specific time intervals, while sizer model cells divide when they reach a certain size. In the adder model, cells divide after a specific amount of growth regardless of initial size and can continue to increase in size across cell divisions. The adder model best fits what is seen in the cambial zone, where daughter cells (also referred to as xylem mother cells) are similar in size, although daughters directly born from the fusiform initials can be smaller than older daughter cells in their final round of division prior to differentiation. However, these models do not account for observed anatomical features of wood such as the large difference in final size of fiber cells compared to vessel elements derived from the same pool of initials, and they do not account for the large variation observed in vessel element size across developmental age, height position within the stem, or in response to environmental cues and stress. Also, these models were developed for cell types that continue dividing, whereas vessel elements undergo terminal differentiation. Although speculative, we favor a model by which an interplay between division rates in the cambial zone and time of onset of differentiation may have an effect on final vessel size, which is manifest or modified through either the time allotted for differentiation or intensity of increase in turgor and cell wall modifications. The interplay of cell division and vessel size can be seen through histological examination of poplar stems from fast growing trees in well-watered conditions, vs trees under water stress. In the former, rapid cell division results in an expanded cambial zone which ultimately produces larger diameter vessel elements. In the latter, slower division rates are associated with a narrow cambial zone that produces smaller diameter vessel elements. Our results showed evidence for enrichment in genes associated with cell cycle and ubiquitination in modules associated with vessel patterning and size traits (**Figures 2**, **3**). Three of the candidate genes within previously identified dQTL for vessel traits (**Table 3**) encode ubiquitin E3 ligases that have been implicated in auxin-mediated cell cycle regulation (SKP2: Jurado et al., 2010), putative morphological and developmental regulation (MAKORIN-like: Arumugam et al., 2007) and organ size (BIG BROTHER: Disch et al., 2006; Cattaneo and Hardtke, 2017) in Arabidopsis.

The current study was limited by several factors. On a practical basis, our sample sizes were modest, and our experimental design did not include stress or other treatments that might allow further dissection of traits and breaking of correlations among traits. A limiting factor was the extremely laborious task of sectioning, imaging, and analyzing images of wood forming tissue from large numbers of stems. Future studies using advanced phenotyping platforms and imaging technologies could no doubt greatly extend the results here. Additionally, our study relied primarily on gene expression data, and undoubtedly key regulatory point of the rapid developmental changes associated with vessel traits include posttranscriptional regulation not surveyed here, including posttranslational modification and protein degradation. However, our study show that it is possible to dissect vessel traits *de novo* and pointed to new candidate mechanisms and genes for future study.

## Data availability statement

The datasets presented in this study can be found in online repositories. The names of the repository/repositories and accession number(s) can be found below: https://www.ncbi.nlm.nih.gov/, PRJNA1061647.

## Author contributions

FR: Investigation, Writing – original draft, Data curation, Formal analysis, Software. ML: Data curation, Formal analysis, Software, Writing – review & editing. AG: Conceptualization, Funding acquisition, Investigation, Methodology, Project administration, Supervision, Writing – original draft.

## References

[B1] Ali O. Cheddadi I. Landrein B. Long Y. (2023). Revisiting the relationship between turgor pressure and plant cell growth. New Phytol. 238, 62–69. doi: 10.1111/nph.18683 36527246

[B2] Anand L. Rodriguez Lopez C. (2022). Chromo Map: an R package for interactive visualization of multi-omics data and annotation of chromosomes. BMC Bioinform. 23, 33. doi: 10.1186/s12859-021-04556-z PMC8753883 35016614

[B3] Anders S. Pyl P. Huber W. (2015). HTSeq, a python framework to work with high-throughput sequencing data. Bioinformatics 31, 166–169. doi: 10.1093/bioinformatics/btu638 25260700 PMC4287950

[B4] Arend M. Fromm J. (2007). Seasonal change in the drought response of wood cell development in poplar. Tree Physiol. 27, 985–992. doi: 10.1093/treephys/27.7.985 17403651

[B5] Arumugam T. U. Davies E. Morita E. H. Abe S. (2007). Sequence, expression and tissue localization of a gene encoding a makorin RING zinc-finger protein in germinating rice (Oryza sativa L. ssp. Japonica) seeds. Plant Physiol. Biochem. 45, 767–780. doi: 10.1016/j.plaphy.2007.07.006 17870591

[B6] Baas P. Ewers F. Davis S. Wheeler E. (2004). “Evolution of xylem physiology,” in Evolution of Plant Physiology. Eds. Hemsley A. Poole I. (Elsevier Academic Press, Cambridge, MA), 273–295.

[B7] Baas P. Werker E. Fahn A. (1983). Some ecological trends in vessel characters. IAWA Bull. New Ser. 4, 141–159.

[B8] Bastiaanse H. Henry I. Tsai H. Lieberman M. Canning C. Comai L.. (2021). A systems genetics approach to deciphering the effect of dosage variation of leaf morphology in Populus. Plant Cell 33, 940–960. doi: 10.1093/plcell/koaa016 33793772 PMC8226299

[B9] Bastiaanse H. Zinkgraf M. Canning C. Tsai H. Lieberman M. Comai L.. (2019). A comprehensive genomic scan reveals gene dosage balance impacts on quantitative traits in *Populus* trees. Proc. Natl. Acad. Sci. 116, 13690–13699. doi: 10.1073/pnas.1903229116 31213538 PMC6613180

[B10] Brodribb T. Field T. (2000). Stem hydraulic supply is linked to leaf photosynthetic capacity: evidence from New Caledonian and Tasmanian rainforests. Plant Cell Environ. 23, 1381–1388. doi: 10.1046/j.1365-3040.2000.00647.x

[B11] Carlquist S. (1984). Vessel grouping in dicotyledon wood. Aliso 10, 505–525. doi: 10.5642/aliso

[B12] Cattaneo P. Hardtke C. (2017). BIG BROTHER uncouples cell proliferation from elongation in the arabidopsis primary root. Plant Cell Physl. 58, 1519–1527. doi: 10.1093/pcp/pcx091 PMC5914324 28922745

[B13] Chaudhary A. Hsiao Y. C. Jessica Yeh F. L. Wu H. M. Cheung A. Y. Xu S. L.. (2023). Brassinosteroid recruits FERONIA to safeguard cell expansion in Arabidopsis. bioRxiv. doi: 10.1101/2023.10.01.560400. [Preprint].

[B14] Chen Z. Hong X. Zhang H. Wang Y. Li X. Zhu J.. (2005). Disruption of the cellulose synthase gene, AtCesA8/IRX1, enhances drought and osmotic stress tolerance in arabidopsis. Plant J. 43, 273–283. doi: 10.1111/j.1365-313X.2005.02452.x 15998313

[B15] D’Ario M. Sablowski R. (2019). Cell size control in plants. Annu. Rev. Genet. 53, 45–65. doi: 10.1146/annurev-genet-112618-043602 31430180

[B16] de Ollas C. Arbona V. GóMez-Cadenas A. (2015). Jasmonoyl isoleucine accumulation is needed for abscisic acid build-up in roots of Arabidopsis under water stress conditions. Plant Cell Environ. 38, 2157–2170. doi: 10.1111/pce.12536 25789569

[B17] Disch S. Anastasiou E. Sharma V. K. Laux T. Fletcher J. C. Lenhard M. (2006). The E3 ubiquitin ligase BIG BROTHER controls arabidopsis organ size in a dosage-dependent manner. Curr. Biol. 16, 272–279. doi: 10.1016/j.cub.2005.12.026 16461280

[B18] Dobin A. Gingeras T. (2015). Mapping RNA-seq reads with STAR. Curr. Protoc. Bioinf. 51, 11.14.1–11.14.19. doi: 10.1002/0471250953.bi1114s51 PMC4631051 26334920

[B19] Esau K. (1977). Anatomy of Seed Plants (New York: Wiley).

[B20] Ewers F. Ewers J. Jacobsen A. López-Portillo J. (2007). Vessel redundancy: modeling safety in numbers. Iawa J. 28, 373. doi: 10.1163/22941932-90001650

[B21] Feng F. Ding F. Tyree M. (2015). Investigations concerning cavitation and frost fatigue in clonal 84K poplar using high-resolution Cavitron measurements. Plant Physiol. 168, 144–155. doi: 10.1104/pp.114.256271 25786827 PMC4424019

[B22] Fonti P. Heller O. Cherubini P. Rigling A. Arend M. (2013). Wood anatomical responses of oak saplings exposed to air warming and soil drought. Plant Biol. 15, 210–219. doi: 10.1111/j.1438-8677.2012.00599.x 22612857

[B23] Gleason S. Westoby M. Jansen S. Choat B. Hacke U. Pratt R. B.. (2016). Weak tradeoff between xylem safety and xylem-specific hydraulic efficiency across the world’s woody plant species. New Phytol. 209, 123–136. doi: 10.1111/nph.13646 26378984

[B24] Guet J. Fichot R. Lédée C. Laurans F. Cochard H. Delzon S.. (2015). Stem xylem resistance to cavitation is related to xylem structure but not to growth and water-use efficiency at the within-population level in Populus nigra L. J. Exp. Bot. 66, 4643–4652. doi: 10.1093/jxb/erv232 25979998

[B25] Hajek P. Leuschner C. Hertel D. Delzon S. Schuldt B. (2014). Trade-offs between xylem hydraulic properties, wood anatomy and yield in Populus. Tree Physiol. 34, 744–756. doi: 10.1093/treephys/tpu048 25009155

[B26] Henry I. Zinkgraf M. Groover A. Comai L. (2015). A system for dosage-based functional genomics in poplar. Plant Cell 27, 2370–2383. doi: 10.1105/tpc.15.00349 26320226 PMC4815095

[B27] Johnson D. Eckart P. Alsamadisi N. Noble H. Martin C. Spicer R. (2018). Polar auxin transport is implicated in vessel differentiation and spatial patterning during secondary growth in Populus. Am. J. Bot. 105, 186–196. doi: 10.1002/ajb2.1035 29578291

[B28] Junghans U. Langenfeld-Heyser R. Polle A. Teichman T. (2004). Effect of auxin transport inhibitors and ethylene on wood anatomy of poplar. Plant Biol. 6, 22–29. doi: 10.1055/s-2003-44712 15095131

[B29] Jurado S. Abraham Z. Manzano C. López-Torrejón G. Pacios L. F. Del Pozo J. C. (2010). The Arabidopsis cell cycle F-box protein SKP2A binds to auxin. Plant Cell 22, 3891–3904. doi: 10.1105/tpc.110.078972 21139066 PMC3027161

[B30] Kubo M. Udagawa M. Nishikubo N. Horiguchi G. Yamaguchi M. Ito J.. (2005). Transcription switches for protoxylem and metaxylem vessel formation. Genes Dev. 19, 1855–1860. doi: 10.1101/gad.1331305 16103214 PMC1186185

[B31] Langfelder P. Horvath S. (2008). WGCNA: an R package for weighted correlation network analysis. BMC Bioinf. 9, 559. doi: 10.1186/1471-2105-9-559 PMC2631488 19114008

[B32] Larson P. (1994). The Vascular Cambium: Development and Structure (Berlin: Springer Verlag). doi: 10.1007/978-3-642-78466-8

[B33] Law C. Chen Y. Shi W. Smyth G. (2014). Voom: precision weights unlock linear model analysis tools for RNA-seq read counts. Genome Biol. 15, R29. doi: 10.1186/gb-2014-15-2-r29 24485249 PMC4053721

[B34] Lens F. Sperry J. Christman M. Choat B. Rabaey D. Jansen S. (2011). Testing hypotheses that link wood anatomy to cavitation resistance and hydraulic conductivity in the genus Acer. New Phytol. 190, 709–723. doi: 10.1111/j.1469-8137.2010.03518.x 21054413

[B35] Li H. Handsaker B. Wysoker A. Fennell T. Ruan J. Homer. (2009). The sequence alignment/map (SAM) format and SAMtools. Bioinformatics 25, 2078–2079. doi: 10.1093/bioinformatics/btp352 19505943 PMC2723002

[B36] Li S. Lin Y. Wang P. Zhang B. Li M. Chen S.. (2019). The AREB1 transcription factor influences histone acetylation to regulate drought responses and tolerance in Populus trichocarpa. Plant Cell 31, 663–686. doi: 10.1105/tpc.18.00437 30538157 PMC6482633

[B37] Liu R. Holik A. Su S. Jansz N. Chen K. Leong H.. (2015). Why weight? Modelling sample and observational level variability improves power in RNA-seq analyses. Nucleic Acids Res. 43, e97. doi: 10.1093/nar/gkv412 25925576 PMC4551905

[B38] Loepfe L. Martinez-Vilalta J. Piñol J. Mencuccini M. (2007). The relevance of xylem network structure for plant hydraulic efficiency and safety. J. Theor. Biol. 247, 788–803. doi: 10.1016/j.jtbi.2007.03.036 17509617

[B39] Olson M. Anfodillo T. Rosell J. Petit G. Crivellaro A. Isnard S.. (2014). Universal hydraulics of the flowering plants: vessel diameter scales with stem length across angiosperm lineages, habits and climates. Ecol. Lett. 17, 988–997. doi: 10.1111/ele.12302 24847972

[B40] Olson M. Pace M. Anfodillo T. (2023). The vulnerability to drought-induced embolism-conduit diameter link: breaching the anatomy-physiology divide. IAWA J. 3-4, 335–354. doi: 10.1163/22941932-bja10123

[B41] Olson M. Rosell J. Martinez-Perez C. Leon-Gomez C. Fajardo A. Isnard S.. (2020). Xylem vessel-diameter–shoot-length scaling: ecological significance of porosity types and other traits. Ecol. Monogr. 90, e01410. doi: 10.1002/ecm.1410

[B42] Olson M. Sorianoa D. Rosell J. Anfodilloc T. Donoghued M. Edwards E.. (2018). Plant height and hydraulic vulnerability to drought and cold. PNAS 115, 7551–7556. doi: 10.1073/pnas.1721728115 29967148 PMC6055177

[B43] Parker G. Schofield R. Sundberg B. Turner S. (2003). Isolation of *COV1*, a gene involved in the regulation of vascular patterning in the stem of Arabidopsis. Development 130, 2139–2148. doi: 10.1242/dev.00441 12668628

[B44] Popko J. Hänsch R. Mendel R.-R. Polle A. Teichmann T. (2010). The role of abscisic acid and auxin in the response of poplar to abiotic stress. Plant Biol. 12, 242–258. doi: 10.1111/j.1438-8677.2009.00305.x 20398232

[B45] Ramachandran P. Augstein F. Mazumdar S. Nguyen T. V. Minina E. A. Melnyk C. W.. (2021). Abscisic acid signaling activates distinct VND transcription factors to promote xylem differentiation in Arabidopsis. Curr. Biol. 31, 3153–3161.e5. doi: 10.1016/j.cub.2021.04.057 34043949

[B46] Ritchie M. Phipson B. Wu D. Hu Y. Law C. Shi W.. (2015). Limma powers differential expression analyses for RNA-sequencing and microarray studies. Nucleic Acids Res. 43, e47. doi: 10.1093/nar/gkv007 25605792 PMC4402510

[B47] Robinson M. McCarthy D. Smyth G. (2010). edgeR: a Bioconductor package for differential expression analysis of digital gene expression data. Bioinformatics 26, 139–140. doi: 10.1093/bioinformatics/btp616 19910308 PMC2796818

[B48] Rodriguez-Zaccaro D. Groover A. (2019). Wood and water: How trees modify wood development to cope with drought. Plants People Planet. 1, 346–355. doi: 10.1002/ppp3.29

[B49] Rodriguez-Zaccaro F. D. Henry I. Groover A. (2021). Genetic regulation of vessel morphology in Populus. Front. Plant Sci. 12. doi: 10.3389/fpls.2021.705596 PMC8419429 34497621

[B50] Scholz A. Rabaey D. Stein A. Cochard H. Smets E. Jansen S. (2013). The evolution and function of vessel and pit characters with respect to cavitation resistance across 10 Prunus species. Tree Physiol. 33, 684–694. doi: 10.1093/treephys/tpt050 23933827

[B51] Shirakawa M. Ueda H. Koumoto Y. Fuji K. Nishiyama C. Kohchi T.. (2014). CONTINUOUS VASCULAR RING (COV1) is a trans-golgi network-localized membrane protein required for Golgi morphology and vacuolar protein sorting. Plant Cell Physiol. 55, 764–772. doi: 10.1093/pcp/pct195 24363287

[B52] Smith M. Fridley J. Yin J. Bauerle T. (2013). Contrasting xylem vessel constraints on hydraulic conductivity between native and non-native woody understory species. Front. Plant Sci. 4, 486. doi: 10.3389/fpls.2013.00486 24348490 PMC3842846

[B53] Smith T. Heger A. Sudbery I. (2017). UMI-tools: modeling sequencing errors in unique molecular identifiers to improve quantification accuracy. Genome Res. 27, 491–499. doi: 10.1101/gr.209601.116 28100584 PMC5340976

[B54] Sperry J. S. (2011). “Hydraulics of vascular water transport,” in Signalling and Communication in Plants: Mechanical Integration of Plant Cells and Plants. Ed. Wojtaszek P. (Springer, Berlin), 303–327.

[B55] Sperry J. Meinzer F. McCulloh K. (2008). Plant Safety and efficiency conflicts in hydraulic architecture: scaling from tissues to trees. Cell Environ. 31, 632–645. doi: 10.1111/j.1365-3040.2007.01765.x 18088335

[B56] Taylor N. G. Howells R. M. Huttly A. K. Vickers K. Turner S. R. (2003). Interactions among three distinct CesA proteins essential for cellulose synthesis. Proc. Natl. Acad. Sci. U.S.A. 100, 1450–1455. doi: 10.1073/pnas.0337628100 12538856 PMC298793

[B57] Tuominen H. Sitbon F. Jacobsson C. Sandberg G. Olsson O. Sundberg B. (1997). Altered growth and wood characteristics in transgenic hybrid aspen expressing Agrobacterium tumefaciens t-DNA indoleacetic acid-biosynthetic genes. Plant Physiol. 109, 1179–1189. doi: 10.1104/pp.109.4.1179 PMC157648 12228661

[B58] Turner S. Gallois P. Brown D. (2007). Tracheary element differentiation. Annu. Rev. Plant Biol. 58, 407–433. doi: 10.1146/annurev.arplant.57.032905.105236 17472568

[B59] Turner S. R. Somerville C. R. (1997). Collapsed xylem phenotype of Arabidopsis identifies mutants deficient in cellulose deposition in the secondary cell wall. Plant Cell. 9, 689–701. doi: 10.1105/tpc.9.5.689 9165747 PMC156949

[B60] Tyree M. Velez V. Dalling J. (1998). Growth dynamics of root and shoot hydraulic conductance in seedlings of five neotropical tree species: scaling to show possible adaptation to differing light regimes. Oecologia 114, 293–298. doi: 10.1007/s004420050450 28307771

[B61] Venturas M. Sperry J. Hacke U. (2017). Plant xylem hydraulics: what we understand, current research, and future challenges. J. Integr. Plant Biol. 59, 356–389. doi: 10.1111/jipb.12534 28296168

[B62] Wilczek A. Wloch W. Iqbal M. Kojs P. (2011). Position of rays and lateral deviation of vessel elements in the stem wood of some dicotyledonous species with storeyed, doublestoreyed, and nonstoreyed cambia. Botany 89, 849–860. doi: 10.1139/b11-074

[B63] Yamaguchi M. Goué N. Igarashi H. Ohtani M. Nakano Y. Mortimer J. C.. (2010). VASCULAR-RELATED NAC-DOMAIN6 and VASCULAR-RELATED NAC-DOMAIN7 effectively induce transdifferentiation into xylem vessel elements under control of an induction system. Plant Physiol. 153, 906–914. doi: 10.1104/pp.110.154013 20488898 PMC2899931

[B64] Yu D. Janz D. Zienkiewicz K. Herrfurth C. Feussner I. Chen S.. (2021). Wood formation under severe drought invokes adjustment of hormonal and transcriptional landscape in poplar. Int. J. Mol. Sci. 22, 9899. doi: 10.3390/ijms22189899 34576062 PMC8493802

[B65] Yu D. Wildhagen H. Tylewicz S. Miskolczi P. Bhalerao P. Polle A. (2019). Abscisic acid signaling mediates biomass trade-off and allocation in poplar. New Phytol. 223, 1192–1203. doi: 10.1111/nph.15878 31050802

[B66] Zhao X. Guo P. Peng H. (2019). An ignored anatomical variable: pore shape shows a nonrandom variation pattern in xylem cross sections. Nordic J. Bot. 37, e02249. doi: 10.1111/njb.02249

