# Supplementary material for: A systems genetic analysis identifies putative mechanisms and candidate genes regulating vessel traits in poplar wood
Source: Front Plant Sci. 2024 May 29;15:1375506. doi: 10.3389/fpls.2024.1375506 (PMC11167656; doi:10.3389/fpls.2024.1375506)
Supplement: Supplementary file 1 [file Table_1.docx]

| **Supplemental Table 1.** List of WGCNA gene modules obtained from a non indel and an indel normalized dataset. Each dataset produced 19 modules. | | | |
| --- | --- | --- | --- |
| **Non indel normalized** | | **Indel normalized** | |
| **Module*** | **Gene number** | **Module*** | **Gene number** |
| Turquoise | 4711 | Turquoise | 4289 |
| Blue | 4630 | Blue | 4267 |
| Brown | 2033 | Lightyellow | 2088 |
| Tan | 1534 | Green | 1488 |
| Darkgreen | 947 | Lightcyan | 1156 |
| Black | 904 | Greenyellow | 997 |
| Pink | 467 | Grey60 | 605 |
| Purple | 358 | Darkorange | 542 |
| Lightgreen | 311 | Lightgreen | 534 |
| Greenyellow | 286 | Tan | 239 |
| Darkturquoise | 285 | Darkred | 239 |
| Cyan | 175 | Salmon | 193 |
| Lightcyan | 147 | Cyan | 188 |
| Grey60 | 144 | Midnightblue | 182 |
| Lightyellow | 137 | Darkgreen | 123 |
| Darkred | 126 | Darkgrey | 97 |
| Darkgrey | 80 | White | 79 |
| Orange | 69 | Steelblue | 54 |
| Darkorange | 60 | Paleturquoise | 46 |
| **Modules with the same color labels are not necessarily equivalent.* | | | |
