# Supplementary material for: A systems genetic analysis identifies putative mechanisms and candidate genes regulating vessel traits in poplar wood
Source: Front Plant Sci. 2024 May 29;15:1375506. doi: 10.3389/fpls.2024.1375506 (PMC11167656; doi:10.3389/fpls.2024.1375506)

**Supplemental Figures**


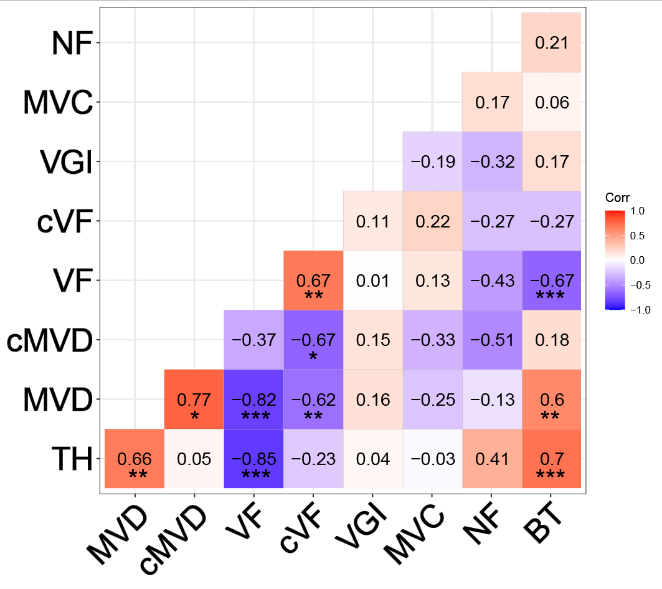


Supplemental Figure 1| Pearson correlation coefficients between different wood and stem traits across 33 hybrid poplar genotypes. Traits include tree height at harvest (TH), mean vessel diameter (MVD), height-corrected mean vessel diameter (cMVD), vessel frequency (VF), height-corrected vessel frequency (cVF), vessel grouping index (VGI), mean vessel circularity (MVC), non-lumen fraction (NF), and bark thickness (BT). Asterisks indicate significant correlations between traits (*p ≤ 0.05; **p ≤ 0.01; ***p ≤ 0.001).


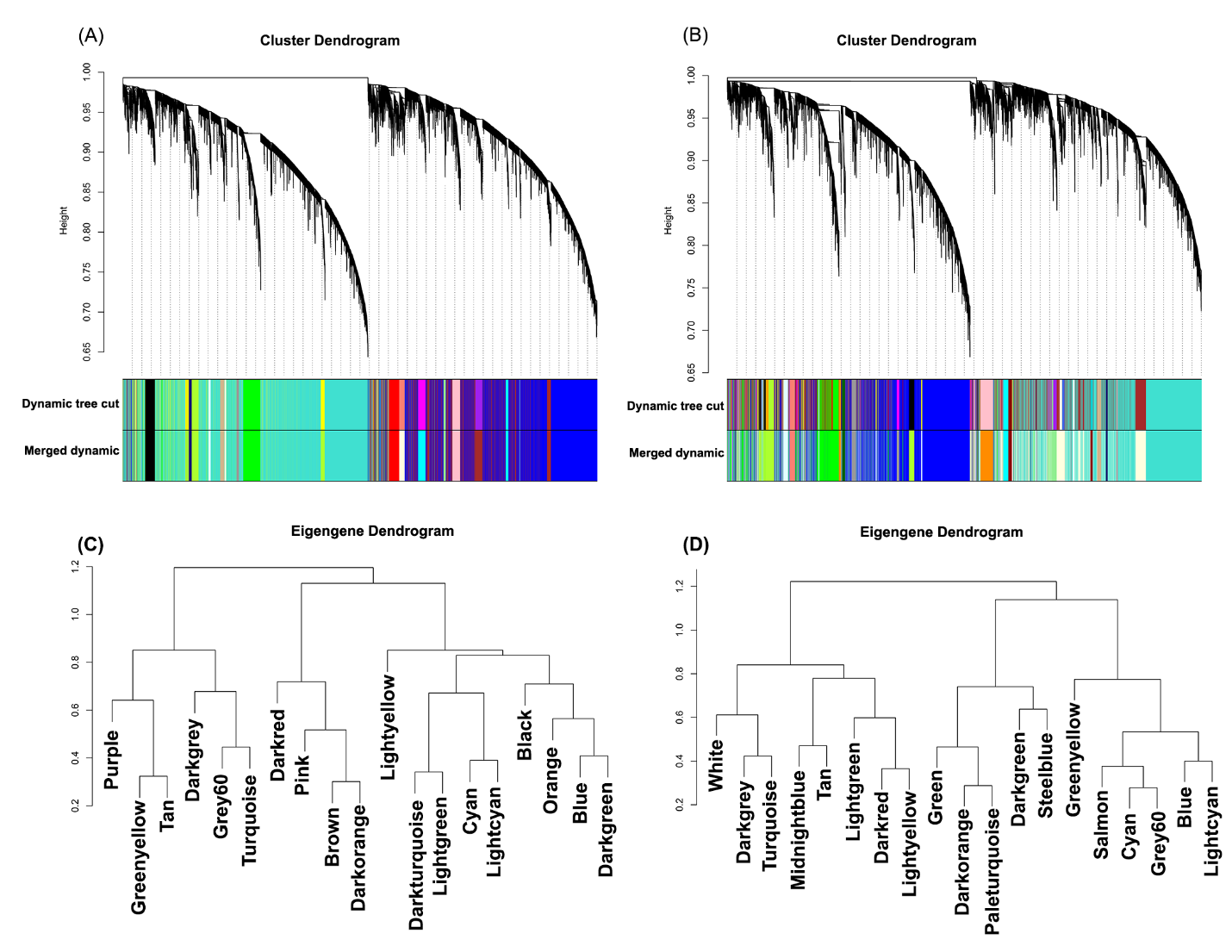


Supplemental Figure 2.| Gene network construction through weighted gene correlation network analysis (WGCNA) using a non indel normalized (A, C) and an indel normalized (B, C) gene counts dataset. Average linkage hierarchical clustering of adjacency-based dissimilarity is represented through dendrograms (A, B), with branches representing groups of highly coexpressed genes. These branches are cut into color-labeled modules based on a fixed height method. Module eigengenes are calculated and used to merge highly correlated modules. Both datasets produced 19 gene modules (C, D).

Supplemental Figure 3.| Stability analyses of gene modules derived from a non indel normalized (A) and an indel normalized (B) gene expression dataset. Dendrogram branches correspond to modules that are represented by color blocks in the row labeled “Full data set”. The color blocks below the first row represent module assignments obtained through repeated resampling of 63% of all sample libraries (49 network iterations constructed). Modules that persist across network construction iterations are considered more stable, or robust.


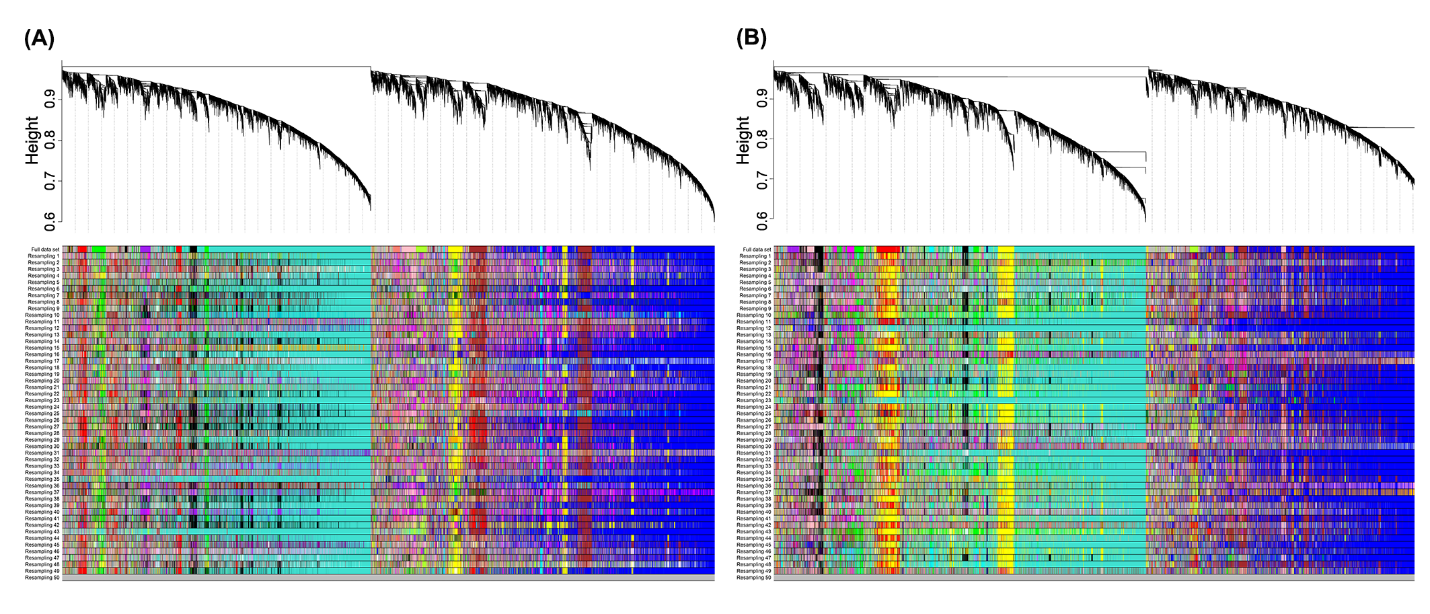

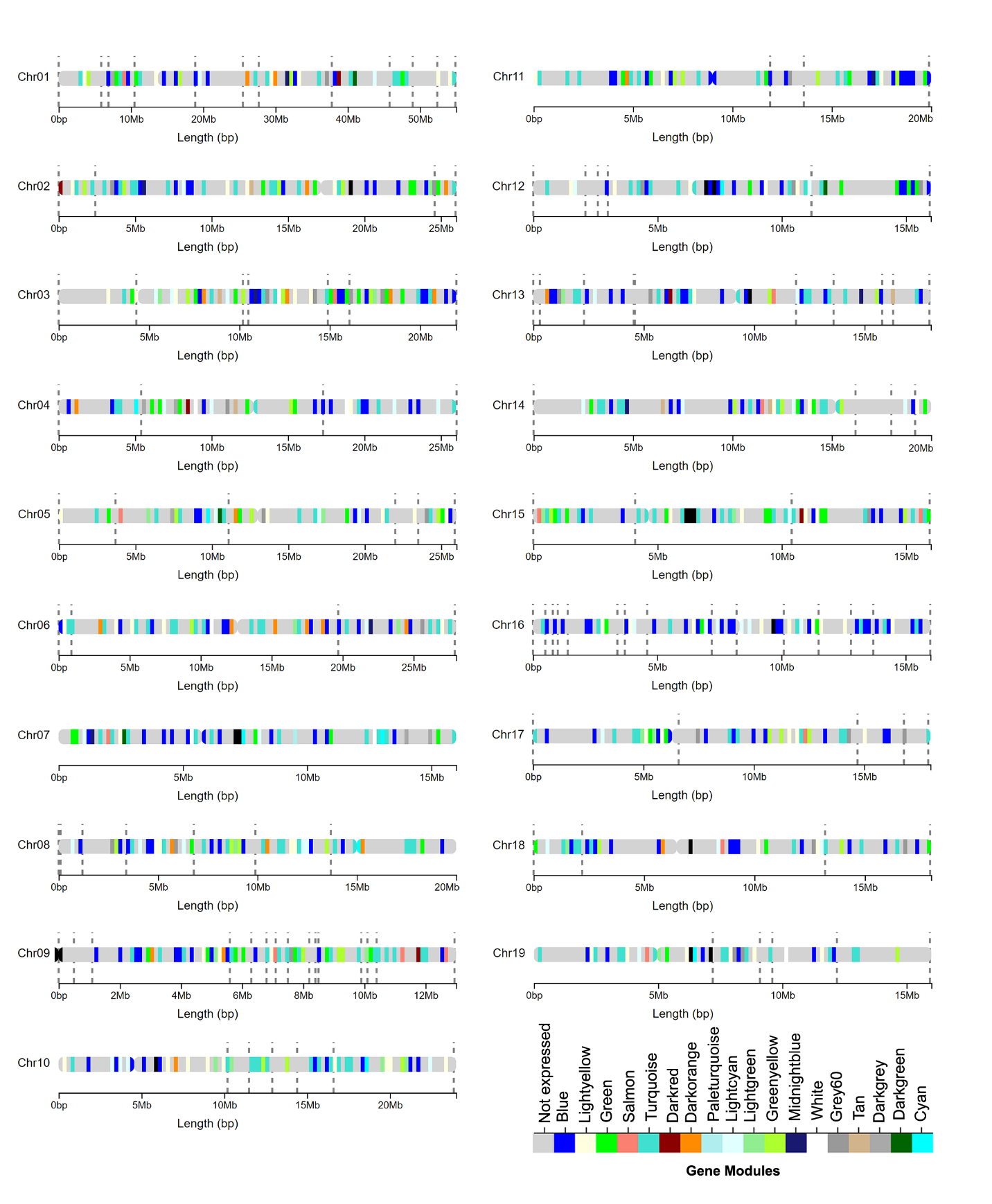


Supplemental Figure 4. Broad genomic locations of gene modules identified through a weighted gene coexpression network analysis (WGCNA) using an indel normalized dataset.

Colored regions along chromosomes represent areas with a high density of genes in a particular module. Gene expression in indel lines at each indel region was replaced by the average expression of all lines at that region. The grey dotted lines mark the edges of indel regions. *The Steelblue module is not shown as it is made up exclusively of genes in unassigned contig scaffolds.


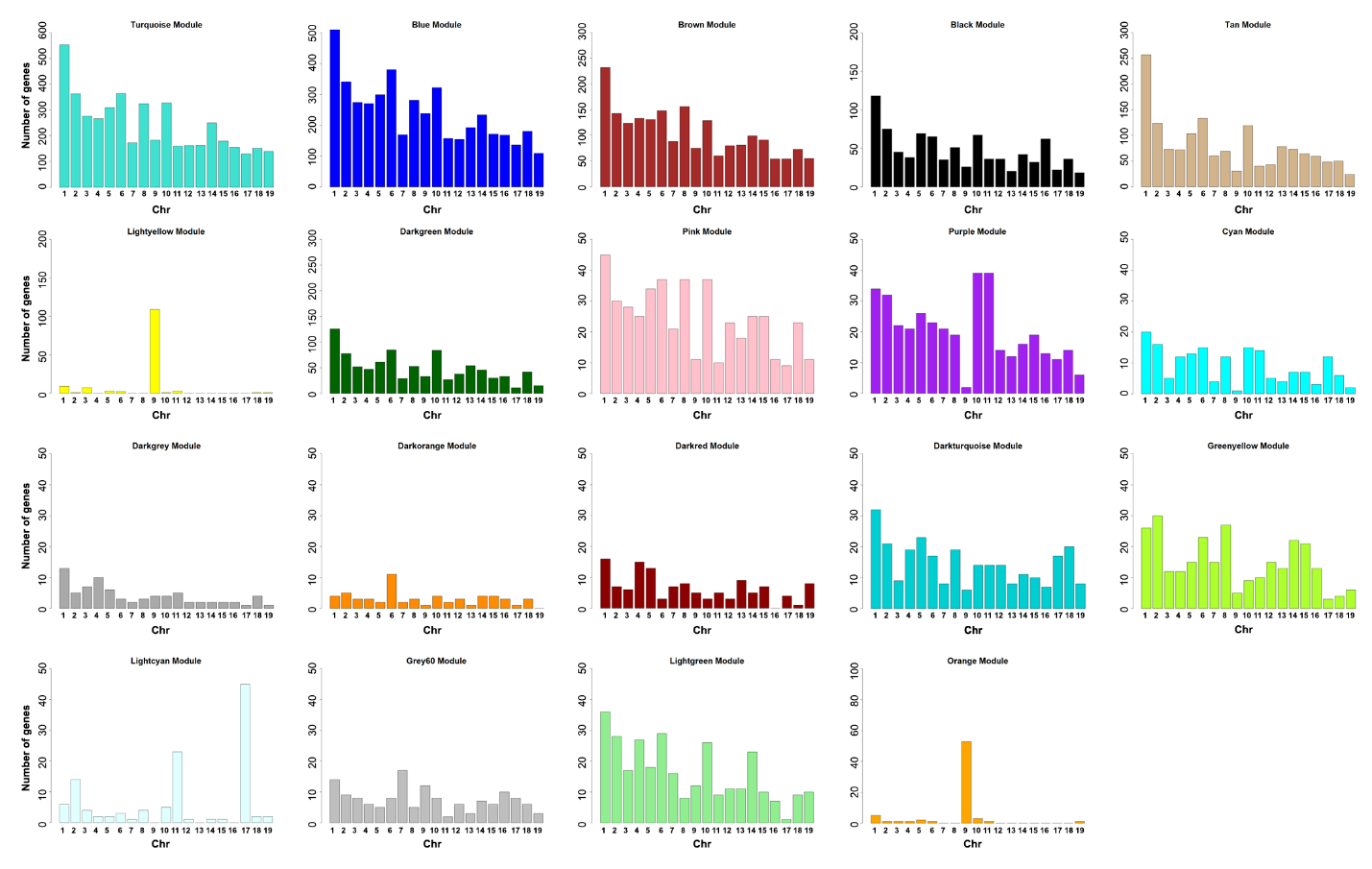


Supplemental Figure 5. Module gene distributions across 19 Poplar chromosomes.

Nineteen modules were generated from a non indel-normalized dataset.

Supplemental Figure 6. Locations and module identities of expressed genes within a previously identified dQTL region in chromosome 9.

Each expressed gene belongs to a particular gene module determined through a WGCNA using a non indel normalized dataset. Seven lines included in the analysis had indel mutations spanning this genomic bin.


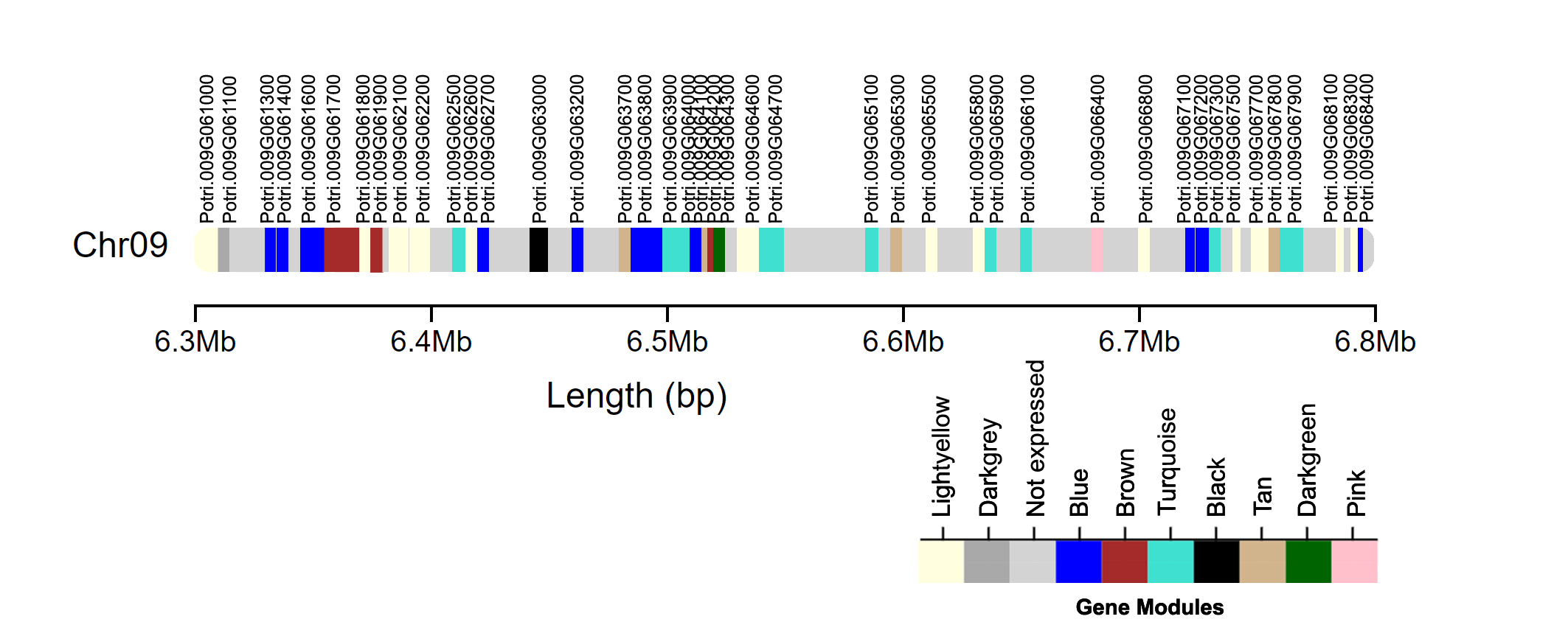


Supplemental Figure 7. Locations and module identities of expressed genes within a previously identified dQTL region in chromosome 16.

Each expressed gene belongs to a particular gene module determined through a WGCNA using a non indel normalized dataset. Six lines included in the analysis had indel mutations spanning this genomic bin.


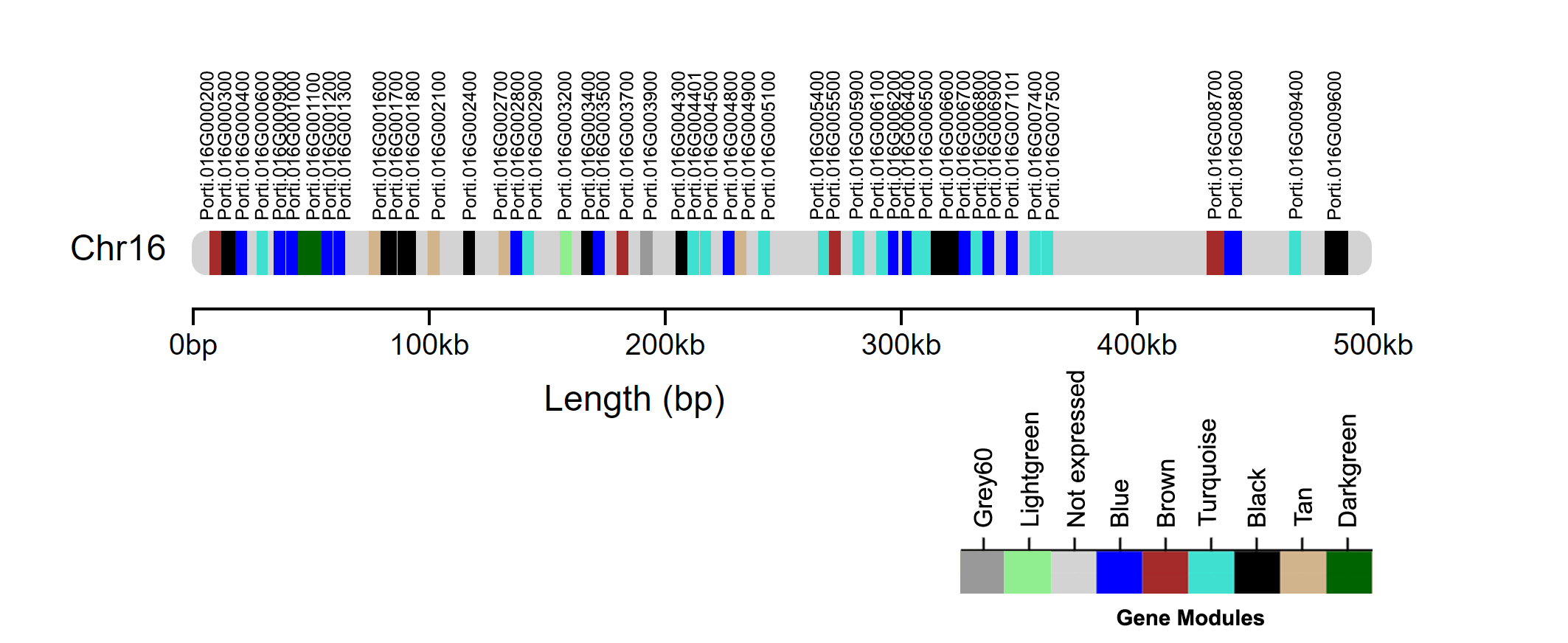

Supplement: Supplementary file 5 [file DataSheet_1.docx]
